# Synthetic Biomimetic Polymethacrylates: Promising Platform for the Design of Anti-Cyanobacterial and Anti-Algal Agents

**DOI:** 10.3390/polym13071025

**Published:** 2021-03-26

**Authors:** Přemysl Mikula, Marie Mlnaříková, Enrico T. Nadres, Haruko Takahashi, Pavel Babica, Kenichi Kuroda, Luděk Bláha, Iva Sovadinová

**Affiliations:** 1 RECETOX, Faculty of Science, Masaryk University, Kamenice 3, CZ-62500 Brno, Czech Republic; premysl.mikula@centrum.cz (P.M.); marie.mlnarikova@recetox.muni.cz (M.M.); pavel.babica@recetox.muni.cz (P.B.); blaha@recetox.muni.cz (L.B.); 2 Department of Biologic and Materials Sciences & Prosthodontics, School of Dentistry, University of Michigan, Ann Arbor, MI 48109, USA; etnadres@gmail.com (E.T.N.); harukot@hiroshima-u.ac.jp (H.T.); kkuroda@umich.edu (K.K.); 3 Department of Experimental Phycology and Ecotoxicology, Institute of Botany of the CAS, CZ-60200 Brno, Czech Republic

**Keywords:** algae, antimicrobials, biomimetic polymers, cyanobacteria, polymethacrylates, water treatment

## Abstract

Extensive, uncontrolled growth of algae and cyanobacteria is an environmental, public health, economic, and technical issue in managing natural and engineered water systems. Synthetic biomimetic polymers have been almost exclusively considered antimicrobial alternatives to conventional antibiotics to treat human bacterial infections. Very little is known about their applicability in an aquatic environment. Here, we introduce synthetic biomimetic polymethacrylates (SBPs) as a cost-effective and chemically facile, flexible platform for designing a new type of agent suitable for controlling and mitigating photosynthetic microorganisms. Since SBPs are cationic and membranolytic in heterotrophic bacteria, we hypothesized they could also interact with negatively charged cyanobacterial or algal cell walls and membranes. We demonstrated that SBPs inhibited the growth of aquatic photosynthetic organisms of concern, i.e., cyanobacteria (*Microcystis aeruginosa* and *Synechococcus elongatus*) and green algae (*Chlamydomonas reinhardtii* and *Desmodesmus quadricauda*), with 50% effective growth-inhibiting concentrations ranging between 95 nM and 6.5 μM. Additionally, SBPs exhibited algicidal effects on *C*. *reinhardtii* and cyanocidal effects on picocyanobacterium *S*. *elongatus* and microcystin-producing cyanobacterium *M*. *aeruginosa*. SBP copolymers, particularly those with moderate hydrophobic content, induced more potent cyanostatic and cyanocidal effects than homopolymers. Thus, biomimetic polymers are a promising platform for the design of anti-cyanobacterial and anti-algal agents for water treatment.

## 1. Introduction

Algae and cyanobacteria are widely distributed in water bodies in the regions with sufficient sunlight, moisture, and nutrients. Their growth is essential in providing oxygen and nutrients for other organisms. However, their excessive propagation is ecological, public health, economic, and technical issues in water management in different societal sectors, including public health, recreation, or industry. Cyanobacteria and algae form blooms in water bodies reducing water quality, which constitutes a hazard concerning the use, safety, and sustainability of drinking and recreational water supplies and fisheries, aquacultures, and agricultural waters [1,2]. Additionally, cyanobacteria and algae accumulate and excessively grow in cooling water industrial systems, which are among the primary problems affecting their efficient operations in many industries, including chemical, petrochemical and food industries, water treatment or power plants [3]. They also colonize surfaces, i.e., form microbial biofilms, by which they can cause biocorrosion, reduce conductive heat transfer across surfaces, and clog hydraulic systems [3,4]. Cyanobacterial and algal blooms and their biofilms have attracted scientific and regulatory attention, and many efforts have been made to prevent, control, and mitigate unwanted cyanobacterial and algal propagation.

Cyanobacterial or algal growth can be inhibited or suppressed by mechanical, physical, biological, or chemical methods [3,5,6,7,8,9,10]. Current chemical methods to control undesired organisms involve various chemicals such as herbicides, flocculating agents, natural products, metals, or photodynamic agents [6,7,9,11]. However, their application is often limited by their non-specificity, low efficiency, and non-target toxicity (reviewed in [9]). For example, metal salts and organometals pose a substantial risk to non-target organisms and an excessive burden on the environment [12]. Herbicide diuron is a general algicide effective in removing phytoplankton but is persistent in the environment and harms non-target biota [9]. Ozone, chlorine, and permanganate are highly effective non-selective algicides used for drinking water and wastewater sanitation with low ecosafety [9].

Antimicrobial synthetic polymers represent a promising tool for fighting microorganisms [13]. The antimicrobial activity against emergency human pathogens has already been demonstrated for different synthetic polymers such as quaternary ammonium, phosphonium, or N-halamine polymers (reviewed in [14,15]). Recently, the concept of cost-effective and biocompatible peptide-mimetic antimicrobial polymers has been introduced [13]. The chemical structure of peptide-mimetic antimicrobial polymers has been inspired by the natural design principle of antimicrobial peptides (host defense peptides). The peptide-mimetic design principle has recently offered significant flexibility and diversity in creating new antimicrobial materials and their potential biomedical applications [13,16,17]. Synthetic biomimetic polymethacrylates (SBPs) are excellent examples of this type of polymers. A cationic amphiphilic nature of these biomimetic polymers is responsible for their antimicrobial activity. This structure was tuned by molecular designing to achieve required antimicrobial activity against a broad spectrum of human pathogenic bacteria, including methicillin-resistant *Staphylococcus aureus*, but cause no harm to mammalian cells [16,18,19]. Notably, these polymers did not result in any measurable resistance development in *Escherichia coli* and displayed high biocompatibility (low or no adverse hemolytic activity). 

Although the antimicrobial activity of peptide-mimetic antimicrobial polymers has already been well-documented, the previous research was almost exclusively focused on human bacterial pathogens to discover novel antibiotics for biomedical applications. Until now, data about their activity against environmentally-relevant target microorganisms have still been limited, which is quite surprising considering the broad spectrum of possible environmental applications of this type of antimicrobial agent. Since SBPs are cationic and membranolytic in heterotrophic bacteria, we hypothesized they could also disrupt negatively charged cyanobacterial or algal cell walls and membranes. Here, we present a systematic screening of cyanostatic/algistatic and cyanocidal/algicidal properties of SBPs against environmentally relevant aquatic photosynthetic microorganisms. To find possible structure-activity relationships and create more potent algicides or cyanocides, we studied structurally different variants focusing on cationic−hydrophobic balance as well as end-group functionality (modified hydrophobic vs. cationic end-group). 

## 2. Materials and Methods

### 2.1. Synthesis of Cationic Amphiphilic Poly(Methacrylate) Derivatives (SBPs)

**Figure 1:**
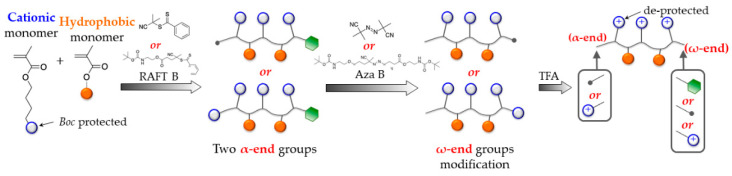
Schematic illustration of the synthesis of synthetic biomimetic polymethacrylates (SBPs).

Methacrylate polymers were synthesized by reversible addition-fragmentation chain transfer (RAFT) polymerization, according to the previous report [20]. The schematic illustration of the synthesis of SBPs is in Figure 1. See Supplementary Materials for further details (Text S1 and Supplementary Table S1). Here the synthesis of **HP1** is described as a representative reaction. For **HP1**, 4-((*tert*-butoxycarbonyl)amino)ethyl methacrylate (ABMA; 4 mmol, 4 mL of 1 M solution in MeCN), 2-cycanopropyl-2-yl-dithiobenzoate (0.6 mmol, 132.6 mg), and AIBN (azobisisobutyronitrile; 0.06 mmol, 9.8 mg) were mixed, and the oxygen of mixture was removed by bubbling with nitrogen for 2 min. The reaction mixture was then stirred at 70 °C for 16 h. The polymer product was isolated by evaporating the MeCN solvent under reduced pressure, and then the residue was dissolved in dichloromethane and precipitated in excess hexanes twice to give *Boc*-**HP1** (*Boc*-protected **HP1**). The mole percentage of EMA (ethyl methacrylate; MP_ethyl_, % of hydrophobic groups) was determined by comparing integrated peaks of butylene groups of *Boc*-ABMA (*boc*-protected ABMA) and ethylene groups of EMA in the ^1^H NMR spectrum (deuterochloroform CDCl_3_ used as a solvent with tetramethylsilane TMS as a stabilizer, 0 ppm). The degree of polymerization (DP) was calculated by comparing integrated peaks of the phenyl group of chain transfer agent at the polymer ω-end and side chains in the ^1^H NMR spectrum. The number average molecular weight (*M_n_*) of the *boc*-protected polymers was calculated using the DP, MP_ethyl_, and the molecular weights of monomers and RAFT agent. The *boc*-protected polymers were also characterized by GPC (gel permeation chromatography) analysis to measure the number average molecular weight (M_n_), and the weight average molecular weight (M_w_) calculated using a calibration curve based on 10 standard samples of poly(methyl methacrylate), MW 500–50,000 (Agilent Technologies, Santa Clara, CA, USA; M-L-10, no. PL2020-0100). 

*Boc*-**HP1** (200 mg) was further mixed with methyl 3-mercaptopropionate (MMP) (100 µL), followed by the addition of trifluoracetic acid (TFA) (8 mL). After stirring for 30 min, the TFA was removed by blowing with nitrogen gas in a closed container connected to a NaOH aq bath to trap TFA gas. The residue was dissolved in methanol and then deprotected **HP1** was isolated by precipitating in excess diethyl ether. Subsequently, the precipitate was dissolved in distilled water and lyophilized to yield a light pink fluffy product (200.7 mg). The *boc*-deprotected polymer was characterized by ^1^H NMR in solvent methanol-d4 CD_3_OD. The ^1^H NMR spectrum was also analyzed using the same method used for the *boc*-protected polymer; DP = 15.2, MP_ethyl_ = 0 mol %. Using these values and molecular weights of monomers and RAFT agent, the M_n_ was calculated (=3900 g/mol, including TFA). GPC analysis was not performed for the *boc*-deprotected polymer due to the low solubility of the *boc*-deprotected polymers in the elution solvent (THF, tetrahydrofuran).

The polymer **HP2** and **CP5** were prepared by transforming the RAFT (dithioester) end group by radical-mediated reaction using AIBN. Due to the lack of phenyl end group, the DP of these polymers cannot be determined by ^1^H NMR analysis. The DP values used for *M_n_* calculations are those of precursor polymers. 

The polymer solution was prepared and diluted in 0.01% acetic acid. A range of final experimental polymer concentrations varied from 0.08 μg mL^−1^ to 40 μg mL^−1^.

### 2.2. Cyanobacterial and Algal Cultures

The laboratory cultures of green algae *Chlamydomonas reinhardtii* (*CR*, CCALA 928) and *Desmodesmus quadricauda* (*DQ*, CCALA 463) and cyanobacterium *Synechococcus elongates* (*SE*, CCALA 187) were purchased from the Culture Collection of Autotrophic Organisms (CCALA, Trebon, Czech Republic). The laboratory culture of cyanobacterium *Microcystis aeruginosa* (*MA*, PCC 7806) was obtained from the Pasteur Culture Collection of Cyanobacteria (Institute Pasteur of Paris, Paris, France). Representative bright-field and fluorescence images of studied freshwater green algae and cyanobacteria species are shown in Figure S1A. All species were continuously cultivated in Erlenmeyer flasks in 50% ZBB medium (freshwater-based medium, pH 7.1, salinity < 0.05%, conductivity 725 μS/cm, Na^+^: 2.3 mM; Ca^2+^: 0.1 mM; Mg^2+^: 0.1 mM), as previously reported [21]. Briefly, the ZBB medium was prepared by mixing the medium Z (ZEHNDER [22]) with the BB medium (Bold’s basal medium [23]) in a ratio of 1:1, autoclaving and subsequent diluting by sterile ultrapure water to 50%. The algae and cyanobacteria were grown under permanent illumination provided by cool white fluorescent tubes (PHILIPS 36 W/33; 1500 l×) at a temperature of 21 ± 1 °C and aerated with sterilized air by passing through 0.22 μm filter (Labicom, Olomouc, Czech Republic). Once a week, half of a cell suspension volume was replaced by the fresh sterile ZBB medium. The population doubling time under the cultivation conditions was 19 h for *CR*, 26 h for *DQ*, 14 h for *SE*, and 27 h for *MA* [21].

### 2.3. Growth Inhibition Assay

Growth inhibition assay was conducted in a microplate format (96-well plates Greiner Bio-One, Kremsmünster, Austria; cat. no. 655201), as reported previously [21]. Briefly, the initial cell concentration (initial inoculum of cells that reached the exponential growth phase diluted in the ZBB medium) was ca. 1 × 10^7^ cells mL^−1^ for cyanobacteria and 1 × 10^6^ cells mL^−1^ for algae. Optical density at 680 nm (OD680) is directly related to cyanobacterial or algal density (cell number) in culture media. The OD680 was measured using Synergy MX microplate reader (Biotek, Bad Friedrichshall, Germany) at the beginning of each experiment (time = 0 h), 24, 48, and 72 h for faster-growing species *CR* and *SE*, and after 48, 72, and 96 h for slower-growing species *DQ* and *MA* [21]. A typical image of a 96-well plate at the end of the growth inhibition assay is shown in Figure S1B.

### 2.4. Algicidal/Cyanocidal Activity Assay

The viability status of algal or cyanobacterial cells can be assessed by measuring the fluorescence of chlorophylls and other photosynthetic pigments, whereby a lack of fluorescence indicates the irreversible damage and death of the cell connected with the disappearance of fluorescence [24,25,26]. Algicidal/cyanocidal activity assay was based on decreased autofluorescence of algal or cyanobacterial cells below the level of inoculated cells. The assay was done as reported previously [21]. The experimental set-up was the same as for the growth inhibition assay. A decline of cell numbers below initial inoculum indicating algicidal or cyanocidal activity was evaluated by monitoring autofluorescence from photosynthetic pigments (λex = 485 nm/λem = 675 nm for green algae; λex = 590 nm/λem = 675 nm for cyanobacteria) using Synergy MX microplate reader (Biotek) measured at the same time points as an optical density for growth inhibition assay. A typical image of a 96-well plate at the end of the algicidal/cyanocidal activity assay is shown in Figure S1B. 

### 2.5. Data Analysis and Statistics

Non- and solvent-treated cells (final concentrations of acetic acid: 0.001 vol%) were included for each treatment. Solvent did not affect the growth or viability of used cyanobacterial or algal strains compared to non-treated cells. Each experiment was repeated at least three times independently. In the growth inhibition assay, optical reading values were normalized to non-treated cells by dividing the OD680 values of the polymer-exposed cells with that of the control groups (the growth of non-treated cells = 100%) for each exposure time. Cyanobacterial and algal growth at each time point was expressed as a percentage of the given negative control. In the algicidal/cyanocidal activity assay, fluorescence readings were normalized to the autofluorescence of inoculated cells at time = 0 h and data are expressed as a percentage of relative fluorescence units (RFUs) for the initial inoculum. IC_50_ values were calculated using non-linear curve fitting (GraphPad Prism 6 software). The final IC_50_ value was calculated as geometric means of IC_50_ values determined for each independent experiment with 95% CI (confidence interval). Graphs were prepared using SigmaPlot 12.1 (Systat Software, Krakow, Poland). One-way ANOVA for normally distributed data with equal variance or Kruskal-Wallis ANOVA was done in SigmaPlot 12.1. Dunnett’s method (multiple comparisons versus control group) or Tukey’s method (multiple comparisons) were selected as post-hoc tests. *p*-values less than or equal to 0.05 were considered significant.

## 3. Results

### 3.1. Design and Characterization of Studied Biomimetic Polymethacrylates (SBPs)

**Figure 2:**
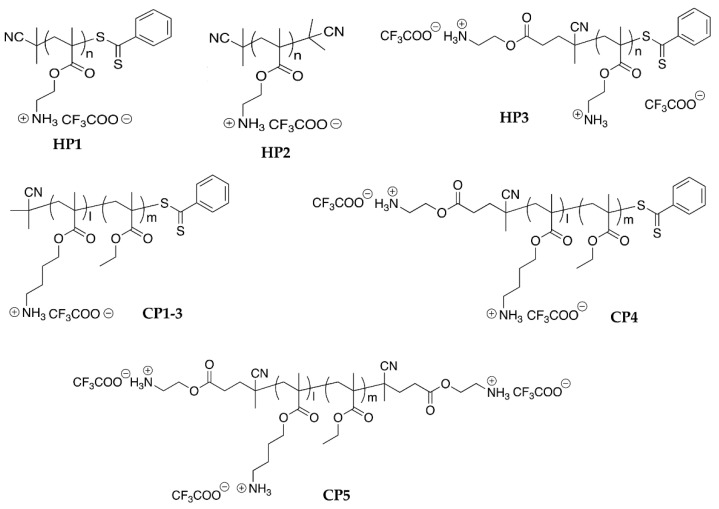
The structure of synthetic biomimetic polymethacrylates (SBPs) studied within this study.

We selected an SBP platform due to providing a set of different structural properties to be tailored. In this study, we tested eight structurally different SBPs to probe their efficacy against cyanobacteria and algae and examine the effect of polymer structures on their antimicrobial activity against them (Table 1, Figure 2 and Table S1). The cationic groups of copolymers provide an electrostatic binding to anionic bacterial or algal cell membranes in the polymer design. The hydrophobic groups are inserted into the hydrophobic domain of the cell membranes to disrupt the membrane, causing leakage of cellular contents and ultimately cell death. Therefore, the polymer structures, net cationic charges, and hydrophobicity would be determined in their activity against cyanobacteria and algae. 

SBPs may contain more hydrophobic groups than conventional polymers, and it may be common to use the minimum content of hydrophobic groups to favor the processing of the material obtained. However, SBPs and their analogs have previously shown potent antimicrobial activity against a broad spectrum of microorganisms and low hemolytic activity against human red blood cells, thereby would be good candidates for environmental applications [20,27]. 

Although the stability of the polymers is beyond the scope of this report, long-term exposure to microbes and the environment may cause polymer degradation. Specifically, it has been known for antimicrobial polymer coatings by acrylic polymers prone to photochemical degradation due to the acrylic hydrogen atom in alpha to the carbonyl group, promoting fungi attack and proliferation on the coatings [28]. However, while the polymers studied here are tested as antimicrobials in solution, not as coatings, they are methacrylate polymers that lack the acrylic hydrogen atom in the alpha position. Therefore, the methacrylate backbone would be relatively stable, and it will be advantageous to provide long-lasting antimicrobial effects in the solution.

The polymers were synthesized by RAFT polymerization to give narrow molecular weight distribution [20]. Three SBPs do not bear hydrophobic groups (homopolymers: **HP1–3**). Five SBPs do bear hydrophobic groups (copolymers) with a different extent (~30–50%: **CP1–5**). Selected polymers were further modified to modulate the end groups (see the synthesis scheme of **HP2** and **CP5** in Text S1B and F) [20]. **CP1**, **CP2**, and **CP3** have the same α- and ω- end groups but were designed to have different compositions of the cationic and hydrophobic groups to examine the effect of the cationic charges and hydrophobicity on their antimicrobial activities. Three SBPs contain the cationic α-end (**HP3**, **CP4**, **CP5**) and five do not (**HP1**, **HP2**, **CP1–3**). SBPs also differ in the ω-end group (phenyl thio-ester: **HP1**, **HP3**, **CP1–4**; cyanoisobutyl: **HP2** and cationic: **CP5**). 

The *boc*-protected polymers were analyzed by GPC to determine the molecular weight distribution. Due to the low solubility of the polymers in the GPC elution solvent (THF), GPC analyses of *boc*-deprotected polymers were not performed. The polydispersity (M*_w_*/M*_n_*) of the *boc*-protected polymers was around 1.1–1.2, indicating the polymers have a relatively narrow molecular weight distribution. The discrepancies between the M*_n_* values determined by ^1^H NMR and those by GPC in Table 1 are likely because (1) the ^1^H NMR M*_n_* values were calculated for the *boc*-deprotected polymers while GPC M_n_ was determined for the *boc*-protected polymers, and (2) the molecular weights measured using GPC are determined relative to the standard samples of polymethylmethacrylate; these cannot be directly compared to the M*_n_* values determined by ^1^H NMR.

### 3.2. Growth-Inhibitory Activity

First, we screened a growth-inhibitory activity of selected SBPs in a 72-h toxicity assay with cyanobacteria *SE* and *MA* and green algae *DQ* and *CR* as model photosynthetic microorganisms by recording optical density as a marker of microbial growth. *SE* and *MA* are common freshwater cyanobacteria and are often utilized as model cyanobacterial species in experimental studies. *MA* produces cyanotoxins (microcystins) and can form harmful algal blooms causing human health and ecological problems [29]. *CR* and *DQ* are ubiquitous eucaryotic freshwater green algae in natural and industrial water systems and can occur in water blooms [30]. Both species are used as a model for antifouling and anti-algal assessments [30,31]. 

**Figure 3:**
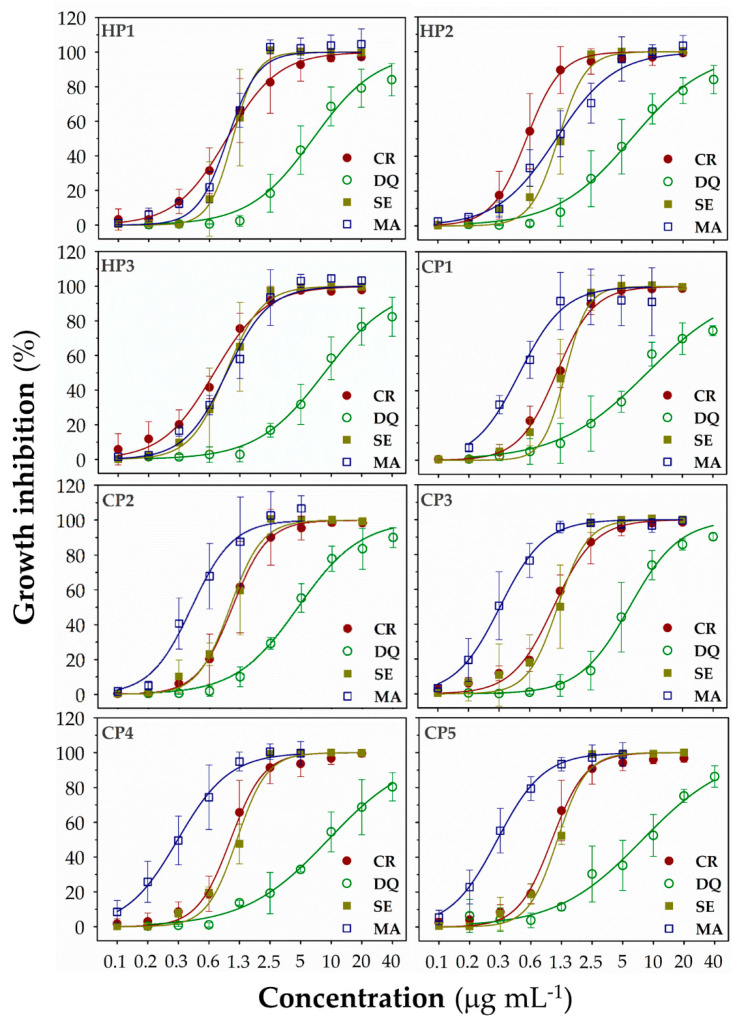
The growth-inhibitory activity of synthetic biomimetic polymethacrylates (SBPs) against freshwater green algae *Chlamydomonas reinhardtii* (*CR*) and *Desmodesmus quadricauda* (*DQ*) and cyanobacteria *Synechococcus elongatus* (*SE*) and *Microcystis aeruginosa* (*MA*). The growth inhibition was measured by optical density at 680 nm after 72-h exposure. The studied polymers were homopolymers (**HP1–3**) and copolymers (**CP1–5**). Data are expressed as a percentage of a non-treated control and presented as means (±SD) of 3–5 independent experiments.

The tested polymers were active against all four autotrophic microorganism species. SBPs caused a concentration-dependent decrease of their growth (Figure 3) with the _72h_IC_50_ (the concentration of polymer causing a 50% inhibition of the cyanobacterial or algal growth after a 72-h exposure) values ranging from 0.2 to 9.1 μg mL^−1^ (Table S2). All SBPs inhibited *CR* and *SE* growth within a relatively narrow concentration range (_72h_IC_50_: 0.6–1.2 μg mL^−1^) and were less efficient against *DQ* (about ten times higher IC_50_ values; _72h_IC_50_: 4.9–9.1 μg mL^−1^). The most sensitive species was *MA*, with the minimal _72h_IC_50_ values up to three times lower than for *SE* and *CR* (_72h_IC_50_: 0.2–1.1 μg mL^−1^). 

We did not observe strong structure-dependent effects of SBPs against algae *CR* and *DQ* and cyanobacterium *SE*, i.e., all polymers inhibited the growth of these algal and cyanobacterial species with similar intensity (Figure 3 and Table S2). In contrast, copolymers were active against cyanobacterium *MA* at lower concentrations than homopolymers, suggesting that efficiency against *MA* improved with increasing hydrophobicity. 

**Figure 4:**
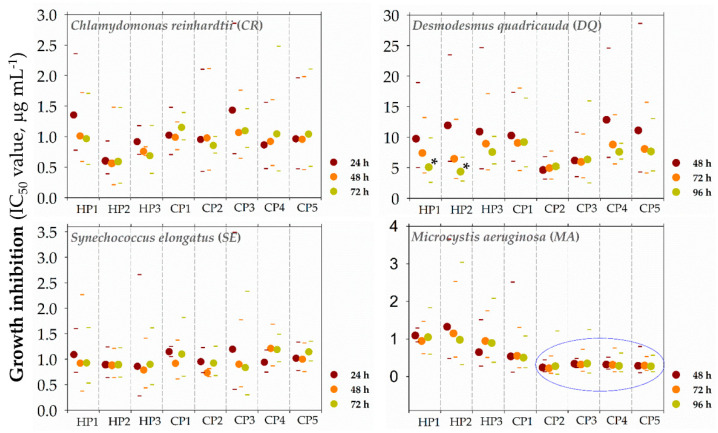
The time-dependency of growth-inhibitory activity of synthetic biomimetic polymethacrylates (SBPs) against freshwater green algae *Chlamydomonas reinhardtii* (CR) and *Desmodesmus quadricauda* (*DQ*) and cyanobacteria *Synechococcus elongatus* (*SE*) and *Microcystis aeruginosa* (*MA*). The growth inhibition was measured by optical density at 680 nm after 24–72 h (CR, *SE*) or 48–96 h (*DQ*, *MA*) exposures. The studied polymers were homopolymers (**HP1–3**) and copolymers (**CP1–5**). Data are expressed as a percentage of a non-treated control and presented as means (±SD) of 3–5 independent experiments. Data are presented as geometric means of IC_50_ values of independent experiments (n = 3–5) with a 95% confidence interval. Asterisk “*” denotes statistically significant differences of IC_50_ value measured after prolonged exposures (48, 72, 96 h) compared to the IC_50_ value after the shortest exposure time (24 h for *CR* and *SE*, 48 h for *DQ* and *MA*) determined by ANOVA with Dunnett’s test (*p* < 0.05). The blue circle marks polymers with profound inhibitory activity against MA, which differs from the rest of polymers (one-way ANOVA with Tukey’s multiple comparison test).

Further, the time-dependency of growth-inhibitory activity was evaluated. We screened polymer activity for 24–72 h or 48–96 h exposures **(Figure 4** and Table S2). The polymers inhibited the growth of the tested microorganisms within the first 24 h (*CR* and *SE*) or 48 h (*DQ* and *MA*). For most polymers, IC_50_ values did not strongly change with increasing exposure duration (72–96 h). Only the activity of homopolymers **HP1** and **HP2** against *DQ* significantly (two-times) increased within time (Figure 3; _48h_IC_50_ = 9.7 and 11.9 vs. _96h_IC_50_ = 5.1 and 4.3 μg mL^−1^, respectively). Anti-algal and anti-cyanobacterial activities were not structure-dependent even after shorter exposure times except for *MA*. Copolymers **CP2–5** with ~30% hydrophobic groups were significantly (almost five times) more active against *MA* after 48 up to 96 h than homopolymers (0% hydrophobic groups), while the effects of the most hydrophobic copolymer **CP1** (47.5% hydrophobic groups) were in between homopolymers and copolymers **CP2–5**.

### 3.3. Algicidal/Cyanocidal Activity

**Figure 5:**
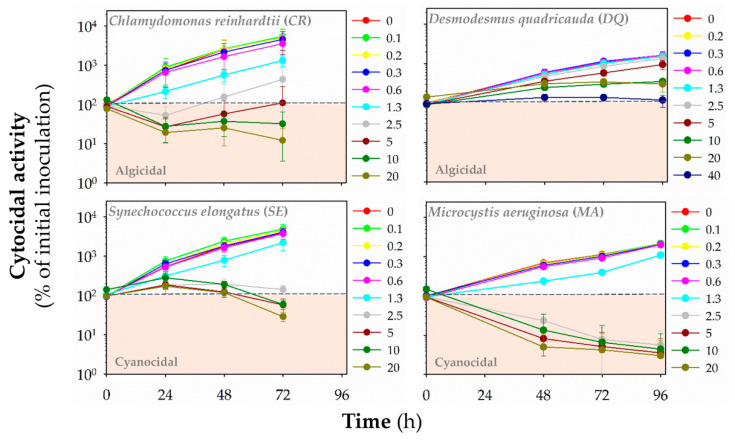
The cytocidal activity of homopolymer **HP1** against freshwater green algae *Chlamydomonas reinhardtii* (*CR*) and *Desmodesmus quadricauda* (*DQ*) and cyanobacteria *Synechococcus elongatus* (*SE*) and *Microcystis aeruginosa* (*MA*). The time- and concentration-dependent effects of the polymers in the range of concentrations of 0–40 μg mL^−1^ on the initial cell number (inoculum) was assessed by red fluorescence (λex = 485 nm/λex = 675 nm for algae, λex = 590 nm/λex = 675 nm for cyanobacteria) at different time points: 24–72-h (*CR*, *SE*) or 48–96-h (*DQ*, *MA*). Data are expressed as a percentage of fluorescence units (RFUs) for initial inoculum and presented as means (±SD) of 3–5 independent experiments.

**Figure 6:**
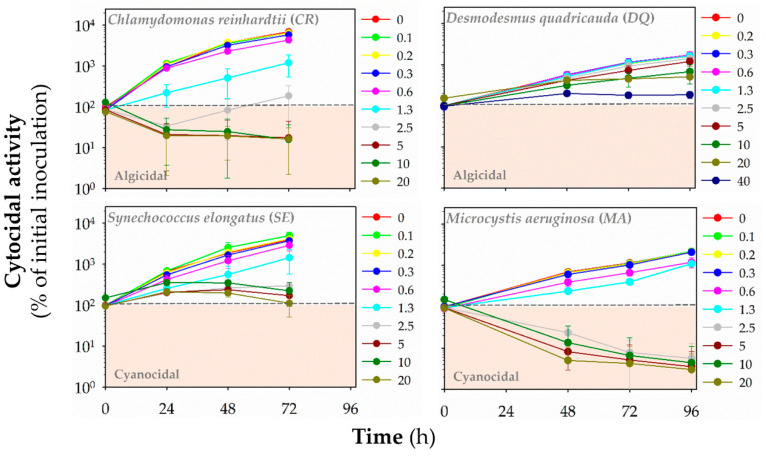
The cytocidal activity of homopolymer **HP3** against freshwater green algae *Chlamydomonas reinhardtii* (*CR*) and *Desmodesmus quadricauda* (*DQ*) and cyanobacteria *Synechococcus elongatus* (*SE*) and *Microcystis aeruginosa* (*MA*). The time- and concentration-dependent effects of the polymers in the range of concentrations of 0–40 μg mL^−1^ on the initial cell number (inoculum) was assessed by red fluorescence (λex = 485 nm/λex = 675 nm for algae, λex = 590 nm/λex = 675 nm for cyanobacteria) at different time points: 24–72 h (*CR*, *SE*) or 48–96 h (*DQ*, *MA*). Data are expressed as a percentage of fluorescence units (RFUs) for initial inoculum and presented as means (±SD) of 3–5 independent experiments.

**Figure 7:**
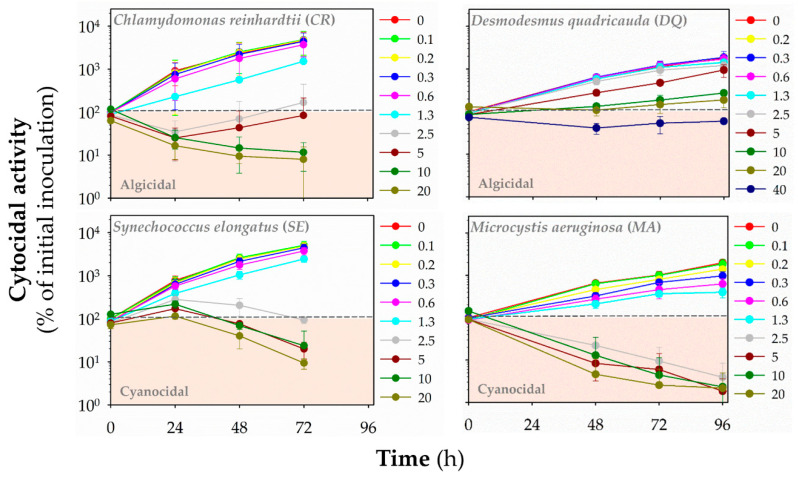
The cytocidal activity of copolymer **CP2** against freshwater green algae *Chlamydomonas reinhardtii* (*CR*) and *Desmodesmus quadricauda* (*DQ*) and cyanobacteria *Synechococcus elongatus* (*SE*) and *Microcystis aeruginosa* (*MA*). The time- and concentration-dependent effects of the polymers in the range of concentrations of 0–40 μg mL^−1^ on the initial cell number (inoculum) was assessed by red fluorescence (λex = 485 nm/λex = 675 nm for algae, λex = 590 nm/λex = 675 nm for cyanobacteria) at different time points: 24–72 h (*CR*, *SE*) or 48–96 h (*DQ*, *MA*). Data are expressed as a percentage of fluorescence units (RFUs) for initial inoculum and presented as means (±SD) of 3–5 independent experiments.

**Figure 8:**
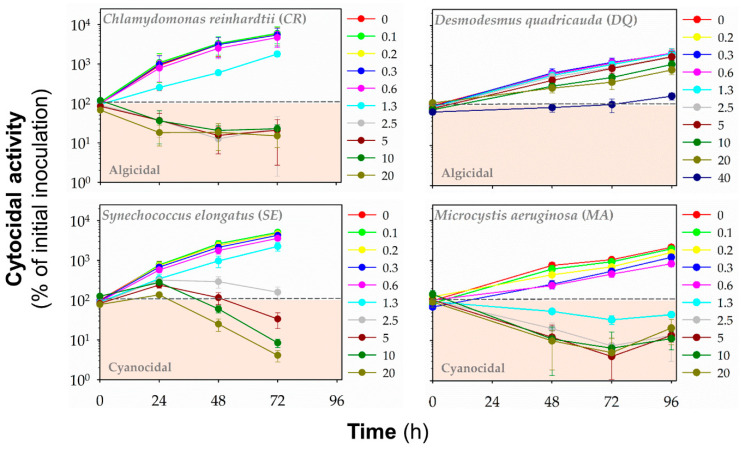
The cytocidal activity of copolymer **CP4** against freshwater green algae *Chlamydomonas reinhardtii* (*CR*) and *Desmodesmus quadricauda* (*DQ*) and cyanobacteria *Synechococcus elongatus* (*SE*) and *Microcystis aeruginosa* (*MA*). The time- and concentration-dependent effects of the polymers in the range of concentrations of 0–40 μg mL^−1^ on the initial cell number (inoculum) was assessed by red fluorescence (λex = 485 nm/λex = 675 nm for algae, λex = 590 nm/λex = 675 nm for cyanobacteria) at different time points: 24–72 h (*CR*, *SE*) or 48–96 h (*DQ*, *MA*). Data are expressed as a percentage of fluorescence units (RFUs) for initial inoculum and presented as means (±SD) of 3–5 independent experiments.

Additionally, we evaluated the cyanocidal and algicidal activities of polymers by monitoring the autofluorescence of chlorophylls and other photosynthetic pigments (**HP1**: Figure 5, **HP2**: Figure S2, **HP3**: Figure 6, **CP1**: Figure S3, **CP2**: Figure 7, **CP3**: Figure S4, **CP4**: Figure 8, **CP5**: Figure S5). Based on fluorescence reading, we could determine whether the polymer decreased the initial (inoculated) number of cells and exhibited algicidal or cyanocidal activities. SBPs could not decrease fluorescence values below the initial inoculum of *DQ*, even at the highest concentration (40 μg mL^−1^) up to 96 h. On the other hand, all homopolymers and copolymers decreased the autofluorescence of *CR* significantly below the autofluorescence of inoculated algal cells within the first 24 h at the concentrations of ≥5 μg mL^−1^ that were higher than those causing the growth inhibition. In contrast, the cyanocidal activity of SBPs against *SE* was structure-dependent. Homopolymers were not active (**HP2** and **HP3**), or their activity was limited (**HP1**). Copolymers decreased a cell number of *SE* below the initial cell numbers in inoculum at the concentration of 5 μg mL^−1^ and higher after the prolonged exposure times (48–72 h). The most potent cytocidal activity of SBPs was observed for *MA*. Like *CR*, all types of SBPs decreased the autofluorescence of MA below the level of inoculated algal cells within the first 24 h, but their effects were manifested at lower concentrations than for *CR*: ≥1.3 μg mL^−1^ for copolymers **CP3–5** and ≥2.5 μg mL^−1^ for the other SBPs. Additionally, the effective cyanocidal concentrations for *SE* and *MA* were higher than those causing the growth inhibition.

## 4. Discussion

The extensive growth of microorganisms has become a widespread issue in managing natural or engineered water systems. Suggested methods for preventing, controlling or mitigating unwanted growth of aquatic microorganisms are limited to a few strategies with practical limitations; therefore, new approaches are urgently required [5,6,7,8,9,12]. Although numerous previously published papers suggested synthetic peptide-mimetic antimicrobial polymers can serve as potent, selective antimicrobial agents with low toxicity to non-target organisms [16,18,19,32,33,34], the research was mainly focused on possible applications of biomimetic polymers in medicine. Model human pathogenic bacteria were almost exclusively used as experimental microorganisms. The studies on the anti-algal or anti-cyanobacterial activities of antimicrobial peptides, whose structure and functions are mimicked by the antimicrobial polymers, against aquatic photosynthetic microorganisms are scarce [35,36]. Very little is also known about the potential effects of synthetic biomimetic polymers in the aquatic environment [21]. Therefore, we transferred a successful polymer-based antimicrobial platform from biomedical applications into a water management sector and studied the potential application of synthetic biomimetic polymers to deal with environmental and engineering issues related to the massive growth of photosynthetic microorganisms.

We used a polymethacrylate platform for designing a new anti-algal or cyanobacterial agent because the polymethacrylate platform can be easily tailored towards the optimization of their biological activities [13], and some of them exhibit strong antimicrobial activity against both Gram-negative and Gram-positive bacteria [16,18,33]. Our study demonstrated that SBPs, synthetic biomimetic polymers based on polymethacrylates, promising antibacterial agents in biomedical applications [16], can be proposed as a promising platform for designing a new type of anti-algal or anti–cyanobacterial agents. SBPs were active against environmentally relevant aquatic photosynthetic microorganisms at concentrations comparable to conventional herbicides with the IC_50_ values ranging from 0.2 to 13 μg mL^−1^, i.e., ca. 95 nM to 6.5 μM. For example, the widely used herbicide diuron [9] is active against algae with the IC_50_ values of 10 up to 900 nM [37,38]. The growth-inhibitory activity of SBPs is similar to the activity of another type of synthetic polymers, branched poly(ethylene imine)s (PEIs), with IC_50_ values ranging from 0.2 to 40 μg mL^−1^ [21]. 

Although the clarification of the molecular mechanisms of SBPs is beyond the scope of this study, we hypothesize that cationic SBPs act primarily through an electrostatic attraction by interacting with the negatively charged components of the algal or cyanobacterial cell wall or membranes as proposed for their effects on human pathogenic bacteria or red blood cells [16,18,19,33]. The negatively charged components of the cell wall are the de-protonation of carboxyl or sulfate groups in the algal cell walls [39,40], peptidoglycan in the cyanobacterial cell walls [41] or the extracellular polysaccharide glycocalyx layer surrounding cell walls [42]. SBPs might bind to the bacterial cell surface, increase the permeability of the outer membrane and block cell division, as reported for cathelin-related antimicrobial peptides and their truncated versions [43]. Additionally, cyanobacterial and algal plasma membranes have an amphipathic character and might also interact with cationic compounds such as SBPs through electrostatic interaction, resulting in blocking cell dividing and inhibiting their growth.

SBPs inhibited the growth of picocyanobacterium *SE* (a small unicellular cyanobacterium), toxin-producing cyanobacterium *MA* and the unicellular flagellated freshwater green alga *CR* with similar intensity. However, all studied SBPs exhibited substantial cytocidal activity only against alga *CR* and cyanobacterium *MA*. Namely, SBPs decreased autofluorescence of photosynthetic pigments below the autofluorescence level of the initial inoculum of *CR* and *MA*, suggesting the cytocidal activity against these microorganisms. The cytocidal activity of the studied cationic polymers on *CR* and *MA* may be related to their cationic nature, as demonstrated for their effects on human pathogenic bacteria or red blood cells [16,18,19,33]. Recently, selective algicidal action of modified antimicrobial peptides with membranolytic activity against marine algal species has been reported [36]. SBPs might bind to and possibly cross the cell wall of *CR* and *MA*, subsequently destabilize and permeabilize cell membranes leading to membrane disruption and cell lysis once the concentrations inside the cell are too high [44,45], as reported for cationic peptides [36]. Moreover, SBPs might bind and internalize into treated algal or cyanobacterial cells and interact with intracellular targets. Mitochondria, thylakoid, or chloroplast membranes of cyanobacterial or algal cells are also negatively charged, and SBPs after internalization might affect or disrupt the structure and function of these organelles as proposed mechanisms for cationic PAMAM dendrimers, branched PEIs or antimicrobial peptides HPA3 and HPA3NT3 [21,36,46,47,48]. 

In contrast, copolymers manifested a cyanocidal effect against *SE*, which appeared after prolonged exposure times (72 h) at the highest polymer concentrations (5–20 μg L^−1^). Thus, these results suggest the selectivity of cytocidal activity of SBPs to *CR* and *MA* over picocyanobacterium *SE*. The cytocidal selectivity might be explained by differences in size (biovolume calculated based on geometric models for phytoplankton [49]: *CR*—113–904 μm^3^ and *MA*—22–113 μm^3^ vs. *SE*—0.1–2 μm^3^), cell shape (spherical shape for *CR* and *MA* vs. rod shape for *SE* [49]) or the cell wall and membrane chemical composition [21]. 

Alga *DQ* was more resistant to the SBP action than algae *CR* and cyanobacteria *SE* and *MA*, also reported for branched PEIs [21]. The sensitivity difference in the growth inhibition was one order of magnitude. Additionally, SBPs acted against *DQ* primarily as algistatic but not algicidal agents even at the highest concentrations and after the prolonged exposure times. This difference in species sensitivity to SBPs might be explained by cell wall composition and cellular arrangements varying among the tested species. The cell wall of *DQ* is primarily formed by a robust biopolymer network consisting of hemicellulosic and sporopolleninic layers [50]. This network likely constitutes a primary site for interaction with SBPs; however, simultaneously, a barrier to the entrance of these polymers into the proximity of the plasma membrane and subsequently into *DQ* cells. Glycoproteins (*CR*) [51,52], peptidoglycans (*SE*, *MA*), or lipopolysaccharides (*SE*, *MA*) [41,42,53,54] are the primary building blocks of the cell walls of other studied species. They might be more vulnerable to the actions of SBPs than hemicellulosic and sporopolleninic layers, as also observed for branched PEIs [21]. Moreover, *DQ* cells form coenobia, which are colonies of laterally joined ellipsoidal cells (2–8 cells). Algae *CR* and cyanobacteria *SE* and *MA* are only unicellular cells, not forming colonies under the experimental conditions in our study. Compact coenobium might reduce the possibility for SBPs to access the individual cells and increase the resistance of joined cells to polymer action compared to solitary cells, as suggested for branched PEIs [21].

SBPs were designed to study what is the desired balance of hydrophobic and cationic character to incorporate the minimum amount of hydrophobic content needed to confer anti-algal and anti-cyanobacterial activities as suggested for their activity against bacterial pathogens [13]. To address that, we evaluated structurally different variants of SBPs varying in hydrophobicity and type of end groups to determine the possible structure-activity relationships. Hydrophobicity and the type of end groups did not play a critical role in the effects of SBPs on the growth of green algae *DQ* and *CR*, as homopolymers and copolymers with different hydrophobicities as well as types of end groups induced comparable effects. Also, the algicidal activity of SBPs observed against *CR* was not dependent on their hydrophobicity or the type of end groups. This finding contrasts with the reported activity of these SBPs against bacterial pathogens or red blood cells [16,18,19], critically dependent on the hydrophobicity. However, the similar structure-dependent activity of SBPs was observed against cyanobacteria, where copolymers, particularly those with moderate content of hydrophobic groups (**CP2**–**5**), inhibited the growth of *MA* more effectively than homopolymers.

Similarly, copolymers but not homopolymers induced significant cyanocidal effects against *SE*. Finally, moderately hydrophobic copolymers **CP3–5** exhibited the most pronounced cyanocidal activity against *MA*. Overall, SBPs represent a promising platform that can be further exploited to design biomimetic polymers with improved anti-cyanobacterial and cyanocidal activities and selectivity.

## 5. Conclusions

SBPs have been widely studied for their biomedical applications against relevant human pathogens. Our study implicates that SBPs are a promising platform for developing a new type of anti-cyanobacterial and anti-algal agents applicable in water systems. The effective doses (95–6.5 μM) were very low comparable, e.g., with those of herbicides. The practical aspects of the anti-algal and anti-cyanobacterial polymers in the real application, especially in open systems such as water reservoirs, should be considered. The non-target toxicity of SBPs to other biota living in water reservoirs needs to be evaluated before their applications to water reservoirs. As SBPs are likely stable and slowly biodegradable, this may be considered undesirable in certain situations such open systems. In such conditions, other structural analogs of SBPs such as poly(ester)-poly(methyl methacrylate) copolymers [55] or polymethacrylic acid grafted psyllium [56] might have a different environmental fate. They should be assessed for their anti-algal and anti-cyanobacterial activities. Our further research aims to determine the anti-algal and anti-cyanobacterial potential of SBPs and their environmentally friendly variants under conditions related to their potential applications. External environmental factors, such as temperature, ionic strength, and pH, vary in different natural or industrial waters and might affect SBP potential against photosynthetic microorganisms.

## Figures and Tables

**Table 1:** Structural characterization of studied synthetic biomimetic polymethacrylates (SBPs) (Structures in Figure 2).

SBP	End Group		HG ^1^ (mol. %)	DP ^2^ ^1^H NMR	*M_n_*^3^ (g/mol) ^1^H NMR	*M_n_*^4^ (g/mol) GPC (*boc*-protected polymer)	Đ ^5^ GPC
	α	ω					(*boc*-protected polymer)
**HP1**	AIBN	Phenyl thio-ester	0	15.2	3900	2400	1.15
**HP2**	AIBN	Cyanoisobutyl	0	n.d.	n.d.	2700	1.15
**HP3**	Cationic	Phenyl thio-ester	0	15.7	3600	2400	1.11
**CP1**	AIBN	Phenyl thio-ester	47.5	16.8	3500	2100	1.12
**CP2**	AIBN	Phenyl thio-ester	27.3	16.2	3900	2100	1.14
**CP3**	AIBN	Phenyl thio-ester	32.8	14.1	3400	2100	1.17
**CP4**	Cationic	Phenyl thio-ester	27.6	10.5	2900	2000	1.11
**CP5**	Cationic	Cationic	26.8	n.d.	n.d.	2000	1.11

**^1^** Hydrophobic group; **^2^** The degree of polymerization (the number of monomeric units in a polymer molecule) determined by ^1^H NMR (proton nuclear magnetic resonance); **^3^** The number average molecular weight (*M_n_*) determined by ^1^H NMR (including trifluoroacetate). The molecular weight calibration was based on poly(methyl methacrylate) standards; **^4^** The number average molecular weight (*M_n_*) of *Boc*-protected polymer determined by GPC (gel permeation chromatography). The molecular weight calibration was based on poly(methyl methacrylate) standards; **^5^** Dispersity (molecular weight distribution) calculated as *M_w_*/*M_n_* using *M_w_* (the weight average molecular weight) and *M_n_* values determined by GPC. AIBN, azobisisobutyronitrile; n.d., not determined.

## Data Availability

The data presented in this study are available in [insert article or supplementary material here].

## Supplementary Materials

The following are available online at https://www.mdpi.com/article/10.3390/polym13071025/s1, Text S1: Polymer synthesis, Table S1: Polymer characterization, Table S2: Growth inhibitory effect of SBPs, Figure S1: The cytocidal activity of homopolymer HP2, Figure S2: The cytocidal activity of copolymer CP1, Figure S3: The cytocidal activity of copolymer CP3, Figure S4: The cytocidal activity of copolymer CP5.
