# Supplementary material for: Synthetic Biomimetic Polymethacrylates: Promising Platform for the Design of Anti-Cyanobacterial and Anti-Algal Agents
Source: Polymers (Basel). 2021 Mar 26;13(7):1025. doi: 10.3390/polym13071025 (PMC8036423; doi:10.3390/polym13071025)
Supplement: Supplementary file 1 [file polymers-13-01025-s001.pdf]

polymers

MDPI

Supporting Materials

Article

Synthetic Biomimetic Polymethacrylates: Promising Platform for the Design of Anti-Cyanobacterial and Anti-Algal Agents

Přemysl Mikula [1], Marie Mlnaříková [1], Enrico T. Nadres [2], Haruko Takahashi [2], Pavel Babica [1,3], Kenichi Kuroda [2], Luděk Bláha [1] and Iva Sovadinová [1,*]

[1] RECETOX, Faculty of Science, Masaryk University, Kamenice 3, CZ-62500 Brno, Czech Republic; premysl.mikula@centrum.cz (P.M.); marie.mlnarikova@recetox.muni.cz (M.M.); pavel.babica@recetox.muni.cz (P.B.); blaha@recetox.muni.cz (L.B.)

[2] Department of Biologic and Materials Sciences & Prosthodontics, School of Dentistry, University of Michigan, Ann Arbor, MI 48109, USA; etnadres@gmail.com (E.N.); harukot@hiroshima-u.ac.jp (H.T.); kkuroda@umich.edu (K.K.)

[3] Department of Experimental Phycology and Ecotoxicology, Institute of Botany of the CAS, CZ-60200 Brno, Czech Republic

* Correspondence: iva.sovadinova@recetox.muni.cz; Tel.: +420-549-494-738

CONTENT

SUPPLEMENTARY TEXT

**Text S1**	Polymer synthesis	S2-S7

SUPPLEMENTARY TABLES

**Table S1**	Characterization of *Boc*-protected and deprotected synthetic biomimetic polymethacrylates (SBPs)	S8
**Table S2**	Growth inhibitory effect of synthetic biomimetic polymethacrylate (SBPs)	S9

SUPPLEMENTARY FIGURES

**Figure S1**	Illustrative images of studied cyanobacterial and algal species and a 96-well plate at the end of assays	S10
**Figure S2**	The cytocidal activity of homopolymer HP2	S11
**Figure S3**	The cytocidal activity of copolymer CP1	S12
**Figure S4**	The cytocidal activity of copolymer CP3	S13
**Figure S5**	The cytocidal activity of copolymer CP5	S14

polymers

MDPI

SUPPLEMENTARY TEXT

Supplementary Text S1

Polymer synthesis

4-Amino-1-butanol was purchased from TCI Chemicals. Di-*tert*-Butyl dicarbonate was purchased from Oakwood Chemicals. The 2-cyanoprop-2-yl-dithiobenzoate and the 4-Cyano-4-(phenylcarbonothioylthio)pentanoic acid were purchased from Strem Chemicals. *N,N′*-dicyclohexylcarbodiimide (DCC), dimethyl aminopyridine (DMAP) and methyl 3-mercaptopropionate (MMP) were purchased from Acros Organics. The 2,2′-azobis(2-methylpropionitrile) was purchased from Sigma-Aldrich. Most of the chemicals were used without further purification, except for ethyl methacrylate (EMA), purchased from Sigma-Aldrich and was freshly distilled before use. The trifluoracetic acid (TFA) and the solvents hexanes, dichloromethane, diethyl ether and methanol were purchased from Fisher. Gel permeation chromatography (GPC; a calibration curve based on 10 standard samples of poly(methyl methacrylates) was performed using a Waters 1515 instrument equipped with Waters Styragel (7.8 mm × 300 mm) HR 0.5, HR 1, and HR 4 columns in sequence and detected by a differential refractometer (RI). Gel permeation chromatography (GPC; a calibration curve based on 10 standard samples of poly(methyl methacrylates), was performed using a Waters 1515 pump and RI detector. 4-((*tert*-butoxycarbonyl)amino)ethyl methacrylate and 4-((*tert*-butoxycarbonyl)amino)butyl methacrylate were synthesized according to the previous report (Nadres et al., Journal of Polymer Science Part A: Polymer Chemistry, 55 (2017), 304–312).

A. Synthesis of homopolymer HP1

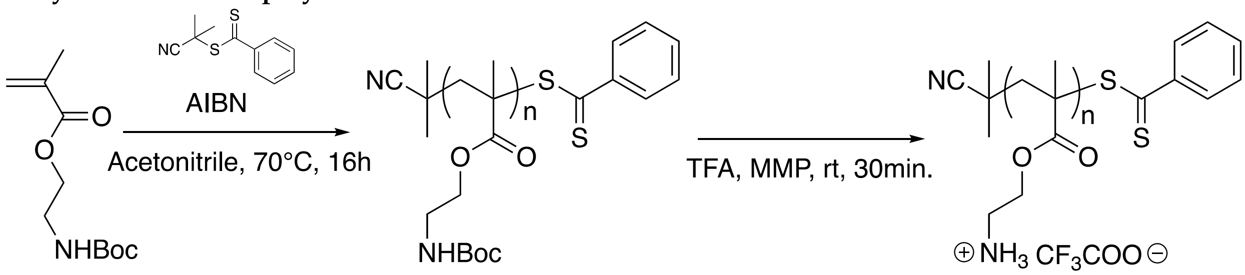

***Boc*-HP1** **HP1**

4-((*tert*-butoxycarbonyl)amino)ethyl methacrylate (4 mmol, 4 ml of 1 M solution in acetonitrile (MeCN)), 2-cycanopropyl-2-yl-dithiobenzoate (0.6 mmol, 132.6 mg), and AIBN (0.06 mmol, 9.8 mg) were mixed in a vial with magnetic stirrer. The oxygen from the mixture was removed by bubbling with nitrogen for 2 min. The reaction mixture was then stirred at 70 °C. After 16 h, the reaction was stopped by cooling the vial in a dry ice-acetone bath. The polymer product was isolated by evaporating the MeCN solvent under reduced pressure, and then the residue was dissolved in dichloromethane and precipitated in excess hexanes twice to give ***Boc*-HP1**. The mol percentage of EMA (MP_{ethyl}) was determined by comparing integrated peaks of butylene groups of *Boc*-ABMA and ethylene groups of EMA in the ^{1}H NMR spectrum. The degree of polymerization (DP) was calculated by comparing integrated peaks of the phenyl group of chain transfer agent at the polymer ω-end and side chains. The number average molecular weight (M_n) was calculated using the DP, MP_{ethyl}, and the molecular weights of monomers and RAFT agent. GPC: M_n = 2400 g/mol, M_w = 2731 g/mol, dispersity ($Đ$) = 1.15. ^{1}H NMR (solvent deuterochloroform $CDCl_3$, with tetramethylsilane TMS as stabilizer , 0 ppm): DP = 10.9, MP_{ethyl} = 0 mol %, M_n = 2700 g/mol.

***Boc*-HP1** (200 mg) was mixed with MMP (100 μL), followed by the addition of TFA (8 mL). After stirring for 30 min, the TFA was removed by blowing with nitrogen gas. The residue was dissolved in methanol and then deprotected **HP1** was isolated by precipitating in excess diethyl ether. Subsequently, the precipitate was dissolved in distilled water and lyophilized to yield a light pink fluffy product (200.7 mg).

^{1}H NMR (solvent methanol-d4 CD_3OD): DP = 15.2, MP_{ethyl} = 0 mol %, M_n = 3900 g/mol (including trifluoroacetate TFA) or 2200 g/mol (excluding TFA).

B. Synthesis of homopolymer HP2

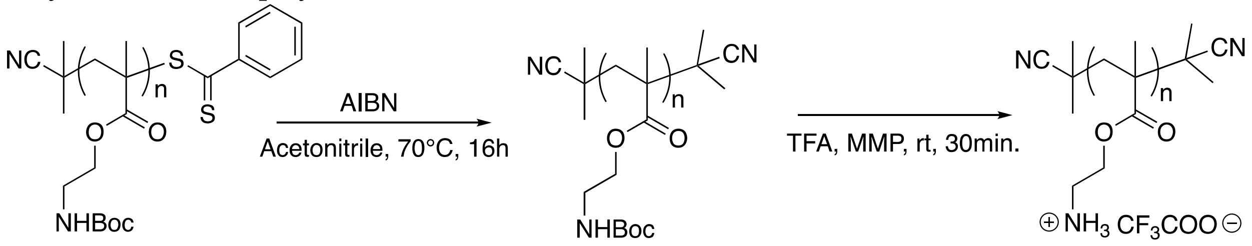

***Boc*-HP1** ***Boc*-HP2** **HP2**

***Boc*-HP1** (200 mg) and AIBN (144 mg, 20 eq) were dissolved in MeCN (120 mL). The solution was bubbled with nitrogen for 1 h and stirred at 70 °C for 16 h. The reaction mixture was cooled to room temperature, and the MeCN was evaporated. The crude product was dissolved in CH_2Cl_2 (2 mL) and purified by precipitation in hexanes (50mL) to give ***Boc*-HP2** (160 mg). GPC: M_n = 2700 g/mol, M_w = 3100 g/mol, $Đ$ = 1.15.

***Boc*-HP2** (123mg) was mixed with MMP (60 μL), followed by the addition of TFA (4.5 mL). After stirring for 30 min, the TFA was removed by blowing with nitrogen gas. The residue was dissolved in methanol and then deprotected **HP2** was isolated by precipitating in excess diethyl ether. Subsequently, the precipitate was dissolved in distilled water and lyophilized to yield a light, fluffy product (105.9 mg, 81%).

C. Synthesis of homopolymer HP3

C-1. Initiator synthesis of bis(2-((*Tert*-butoxycarbonyl)amino)ethyl) (4,4-azobis(4-cyanovalerate)) (Aza B) (Aza B)

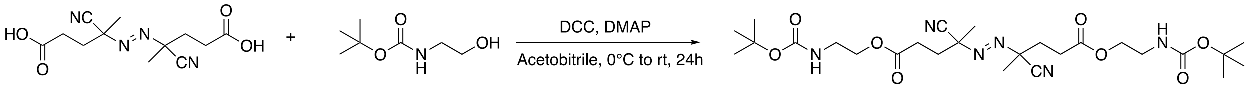

Aza B

tert-Butyl (2-hydroxyethyl)carbamate (25 mmol, 4.08 g), DCC (25 mmol, 5.16 g) and DMAP (2.5 mmol, 306 mg) was dissolve in 50 mL anhydrous MeCN at 0 °C. Then 4,4-azobis(4-cyanovaleric acid) (10 mmol, 2.8 g) was added. The mixture was stirred in the dark at room temperature. After 24 h, the suspension was filtered, and the white solid was washed with cold MeCN (10 mL). The combined filtrate and washings were evaporated, and the residue was dissolved in dichloromethane (50 mL). The resulting solution was washed water (3×), saturated $NaHCO_3$ (3×) and brine (3×), then dried with MgSO4. The residue was loaded to a silica gel column (solvent, 60:40 hexanes-ethyl acetate) to give the purified white solid product (5.40 g, 95 % yield, R_f= 60:40 hexanes-ethyl acetate). The mixture of *dl* and *meso* isomers. ^{1}H NMR ($CDCl_3$, 400 MHz) δ 4.94-4.83 (m, 2H, *k*), 4.20-4.16 (m, 4H, *o*), 3.30-3.40 (m, 4H, *p*), 2.37-2.60 (m, 8H, *m*+*n*), 1.74 (s, 3H, *i*), 1.69 (s, 3H, *i*), 1.45 (s, 18H, *l*). ^{13}C NMR ($CDCl_3$, 400 MHz) δ 171.3, 171.2, 155.8, 117.5, 117.4, 79.6, 71.7, 64.3, 39.4, 33.1, 33.0, 29.0, 28.9, 28.3, 23.9, 23.7.

C-2. RAFT agent synthesis 2-((*Tert*-Butoxycarbonyl)amino)ethyl 4-cyano-4-((phenylcarbonothioyl)thio)pentanoate (RAFT B)

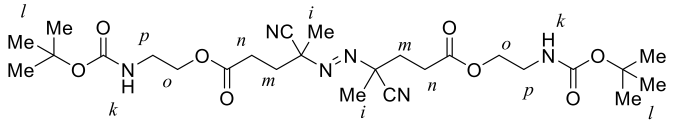

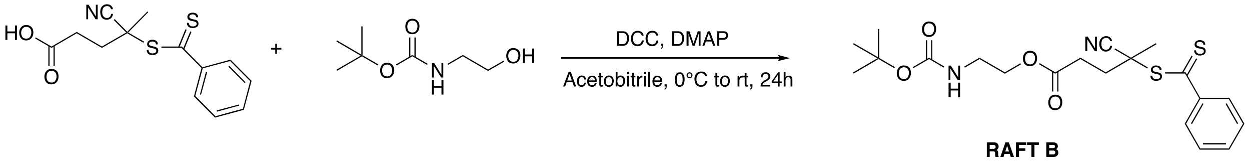

RAFT B

4-Cyano-4-((phenylcarbonothioyl)thio)pentanoic acid (2.5 mmol, 698 mg), *tert*-Butyl (2-hydroxyethyl)carbamate (5.0 mmol, 805 mg), DCC (2.75 mmol, 5.67 mg) and DMAP (2.7 mmol, 33 mg) was dissolve in 25 mL anhydrous MeCN at 0 °C. The mixture was stirred in the dark at room temperature. After 24 h, the suspension was filtered and the white solid was washed with cold MeCN (5 mL). The combined filtrate and washings was evaporated and the residue was loaded to silica gel column (solvent, 70:30 hexanes-ethyl acetate) to give purified white solid product (580 mg, 55 % yield, R_f= 70:30 hexanes-ethyl acetate). ^{1}H NMR ($CDCl_3$, 400 MHz) δ 7.91 (d, *J*=7.6 Hz, 2H, *j*), 7.57 (t, *J*=7.6 Hz, 1H, *j*), 7.40 (d, *J*=7.6 Hz, 2H, *j*), 4.80 (*br* s, 1H, *k*), 4.18 (t, *J*=5.3 Hz, 2H, *o*), 3.39-3.43 (m, 2H, *p*), 2.70-2.66 (m, 2H, *n+m*), 1.94 (s, 3H, *i*), 1.45 (s, 9H, *l*). ^{13}C NMR ($CDCl_3$, 400 MHz) δ 222.2, 171.4, 155.8, 144.5, 133.0, 128.6, 126.6, 118.4, 79.6, 64.3, 45.7, 39.5, 33.3, 29.7, 28.3, 24.2.

C-3. Polymer synthesis HP3

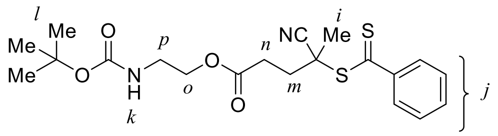

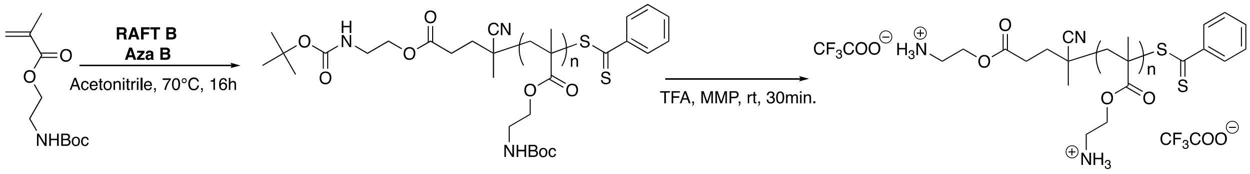

***Boc*-HP3** **HP3**

4-((*tert*-butoxycarbonyl)amino)ethyl methacrylate (5 mmol, 1.15 g), **RAFT B** (0.5 mmol, 211 mg), and **Aza B** (0.05 mmol, 28 mg) were mixed in MeCN (5 mL). The oxygen from the mixture was removed by bubbling with nitrogen for 2 min. The reaction mixture was then stirred at 70 °C. After 16 h, the reaction was stopped by cooling the vial in a dry ice-acetone bath. The polymer product was isolated by evaporating the MeCN solvent under reduced pressure, and then the residue was dissolved in dichloromethane and precipitated in excess hexanes twice to give ***Boc*-HP3**. The DP was calculated by comparing integrated peaks of phenyl group of chain transfer agent at the polymer ω-end and side chains. The number average molecular weight (M_n) was calculated using the DP, MP_{ethyl}, and the molecular weights of monomers and RAFT agent. GPC: M_n = 2400 g/mol, M_w = 2600 g/mol, $Đ$ = 1.11. ^{1}H NMR ($CDCl_3$): DP = 14.0, M_n = 3600 g/mol (including TFA) or 2370 g/mol (excluding TFA).

***Boc*-HP3** (100 mg) was mixed with MMP (50 µL), followed by the addition of TFA (1 mL). After stirring for 30 min, the TFA was removed by blowing with nitrogen gas. The residue was dissolved in methanol and then deprotected **HP3** was isolated by precipitating in excess diethyl ether. Subsequently, the precipitate

was dissolved in distilled water and lyophilized to yield a light pink fluffy product (84.1 mg). ^{1}H NMR (CD_3OD): DP = 15.7, M_n = 3,600 g/mol (including TFA) or 2370 g/mol (excluding TFA).

D. Synthesis of copolymers CP1-3

TFA

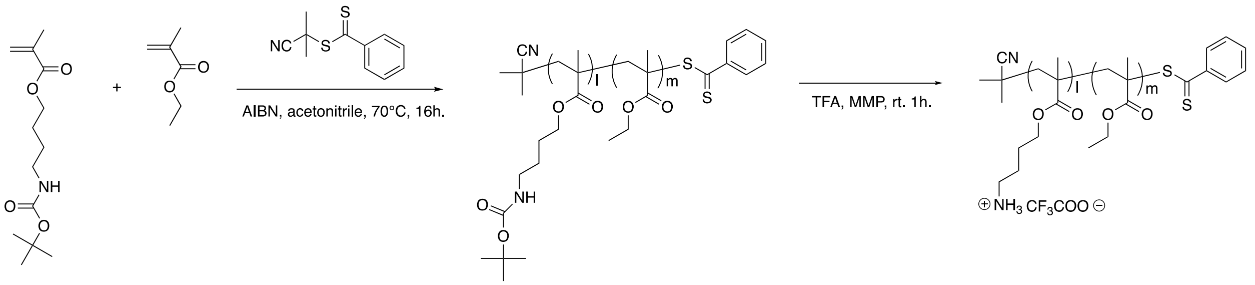

***Boc*-CP1-3** **CP1-3**

D-1. Synthesis of copolymer CP1

4-((*tert*-butoxycarbonyl)amino)butyl methacrylate (5 mmol, 1.29g), EMA (5 mmol, 0.63 mL), 2-cycanopropyl-2-yl-dithiobenzoate (1.5 mmol, 331.86 mg), and AIBN (0.10 mmol, 16.4 mg) were mixed in MeCN (5 mL) with magnetic stirrer. The oxygen from the mixture was removed by bubbling with nitrogen for 2 min. The reaction mixture was then stirred at 70 °C. After 16 h, the reaction was stopped by cooling the vial in a dry ice-acetone bath. The polymer product was isolated by evaporating the MeCN solvent under reduced pressure, and then the residue was dissolved in dichloromethane and precipitated in excess hexanes twice to give ***Boc*-CP1**. The mol percentage of EMA (MP_{ethyl}) was determined by comparing integrated peaks of butylene groups of Boc-ABMA and ethylene groups of EMA in the ^{1}H NMR spectrum. The DP was calculated by comparing integrated peaks of phenyl group of chain transfer agent at the polymer ω-end and side chains. The number average molecular weight (M_n) was calculated using the DP, MP_{ethyl}, and the molecular weights of monomers and RAFT agent. GPC: M_n = 2100 g/mol, M_w = 2350 /mol, Đ = 1.12. ^{1}H NMR ($CDCl_3$): DP = 13.7, MP_{ethyl} = 48.7 mol %, M_n = 2800 g/mol.

***Boc*-CP1** (400 mg) was mixed with MMP (200 μL) in DCM (0.5 mL), followed by the addition of TFA (8 mL). After stirring for 30 min, the TFA was removed by blowing with nitrogen gas. The residue was dissolved in methanol, and then deprotected **CP1** was isolated by precipitating in excess diethyl ether. Subsequently, the precipitate was dissolved in distilled water and lyophilized to yield a light pink fluffy product (329 mg). ^{1}H NMR (CD_3OD): DP = 16.8, MP_{ethyl} = 47.5 mol %, M_n = 3500 g/mol (including TFA) or 2530 g/mol (excluding TFA).

D-2. Synthesis of copolymer CP2

4-((*tert*-butoxycarbonyl)amino)butyl methacrylate (2.8 mmol, 720 mg), EMA (1.2 mmol, 15 μL), 2-cycanopropyl-2-yl-dithiobenzoate (0.6 mmol, 132.8 mg), and AIBN (0.12 mmol, 19.7 mg) in MeCN (2 mL) were used to prepare ***Boc*-CP2** (955 mg). GPC: M_n = 2100 g/mol, M_w = 2390 g/mol, Đ = 1.14. ^{1}H NMR ($CDCl_3$): DP = 15.0, MP_{ethyl} = 29.6 mol %, M_n = 3400 g/mol.

***Boc*-CP2** (200 mg) was mixed with MMP (100 μL), followed by the addition of TFA (4 mL). After stirring for 30 min, the TFA was removed by blowing with nitrogen gas. The residue was dissolved in methanol, and then deprotected **CP2** was isolated by precipitating in excess diethyl ether. Subsequently, the precipitate was dissolved in distilled water and lyophilized to yield a light pink fluffy product (179 mg). ^{1}H NMR (CD_3OD): DP = 16.2, MP_{ethyl} = 27.3 mol %, M_n = 3900 g/mol (including TFA) or 2590 g/mol (excluding TFA).

D-3. Synthesis of copolymer CP3

4-((*tert*-butoxycarbonyl)amino)butyl methacrylate (2.45 mmol, 1.23 mL of 2M solution), EMA (1.05 mmol, 0.53mL of 2M solution), 2-cycanopropyl-2-yl-dithiobenzoate (0.35 mmol, 77 mg), and AIBN (0.035 mmol, 5.74 mg) were used to prepare ***Boc*-CP3** (673 mg, 75%). GPC: M_n = 2100 g/mol, M_w = 2620 /mol, $Đ$ = 1.17. ^{1}H NMR ($CDCl_3$): DP = 14.5, MP_{ethyl} = 35.3 mol %, M_n = 3200 g/mol.

***Boc*-CP3** (100 mg) was mixed with MMP (50 μL), followed by the addition of TFA (1 mL). After stirring for 30 min, the TFA was removed by blowing with nitrogen gas. The residue was dissolved in methanol, and then deprotected **CP3** was isolated by precipitating in excess diethyl ether. Subsequently, the precipitate was dissolved in distilled water and lyophilized to yield a light pink fluffy product (91.3 mg). ^{1}H NMR (CD_3OD): DP = 14.1, MP_{ethyl} = 32.8 mol %, M_n (^{1}H NMR) = 3400 g/mol (including TFA) or 2230 g/mol (excluding TFA).

E. Synthesis of copolymers CP4

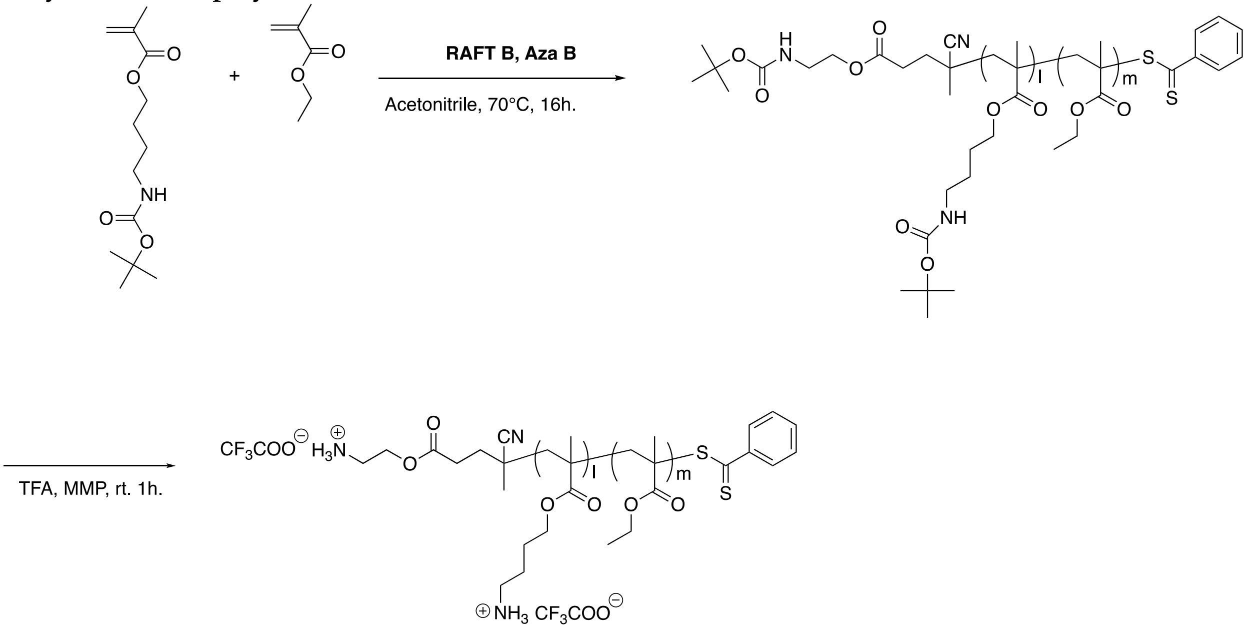

CP4

4-((*tert*-butoxycarbonyl)amino)butyl methacrylate (3.3 mmol, 1.65 mL of 2M solution in MeCN), EMA (1.7 mmol, 0.85 mL of 2M solution in MeCN), **RAFT B** (0.5 mmol, 211 mg), and **Aza B** (0.05 mmol, 28 mg) were mixed in a flask with magnetic stirrer. The oxygen from the mixture was removed by bubbling with nitrogen for 2 min. The reaction mixture was then stirred at 70 °C. After 16 h, the reaction was stopped by cooling the vial in a dry ice-acetone bath. The polymer product was isolated by evaporating the MeCN solvent under reduced pressure, and then the residue was dissolved in dichloromethane and precipitated in excess hexanes twice to give ***Boc*-BC** (780 mg). The mol percentage of EMA (MP_{ethyl}) was determined by comparing integrated peaks of butylene groups of Boc-ABMA and ethylene groups of EMA in the ^{1}H NMR spectrum. The DP was calculated by comparing integrated peaks of phenyl group of chain transfer agent at the polymer ω-end and side chains. The number average molecular weight (M_n) was calculated using the DP, MP_{ethyl}, and the molecular weights of monomers and RAFT agent. GPC: M_n = 2000 g/mol, M_w = 2200 /mol, $Đ$ = 1.11. ^{1}H NMR ($CDCl_3$): DP = 11.1, MP_{ethyl} = 32.3 mol %, M_n = 2800 g/mol.

***Boc*-CP4** (100 mg) was mixed with MMP (50 μL), followed by the addition of TFA (1 mL). After stirring for 30 min, the TFA was removed by blowing with nitrogen gas. The residue was dissolved in methanol, and then deprotected **CP4** was isolated by precipitating in excess diethyl ether. Subsequently, the precipitate

was dissolved in distilled water and lyophilized to yield a light pink fluffy product (86.6 mg). ^{1}H NMR (CD_3OD): DP = 10.5, MP_{ethyl} = 27.6 mol %, M_n = 2900 g/mol (including TFA) or 1860 g/mol (excluding TFA).

F. Synthesis of copolymers CP5

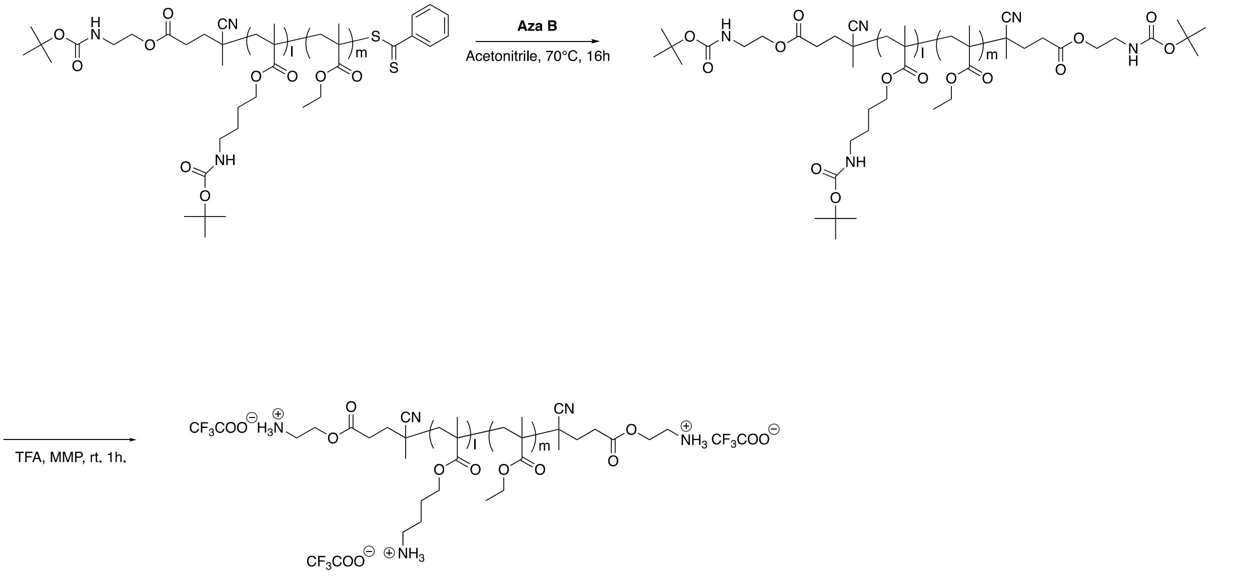

CP5

***Boc*-CP4** (350 mg) and AIBN (1.5 g, 20 eq) were dissolved in MeCN (210 mL). The solution was bubbled with nitrogen for 1 h and stirred at 70 °C for 16 h. The reaction mixture was cooled to room temperature, and the MeCN was evaporated. The crude product was dissolved in CH_2Cl_2 (2 mL) and purified by column chromatography (Sephadex LH20 in methanol) to give ***Boc*-CP5** (235 mg). GPC: M_n = 2000 g/mol, M_w = 2220 g/mol, $Đ$ = 1.11. ^{1}H NMR: MP_{ethyl} = 27.5 mol %.

Boc-CP5 (170 mg) was mixed with MMP (85 µL), followed by the addition of TFA (1.7 mL). After stirring for 30 min, the TFA was removed by blowing with nitrogen gas. The residue was dissolved in methanol, and then deprotected **CP5** was isolated by precipitating in excess diethyl ether. Subsequently, the precipitate was dissolved in distilled water and lyophilized to yield a light, fluffy product (159.7 mg). ^{1}H NMR (CD_3OD): MP_{ethyl} = 26.8 mol %.

polymers

MDPI

SUPPLEMENTARY TABLES

Supplementary Table S1: Characterization of *Boc*-protected and deprotected synthetic biomimetic polymethacrylates (SBPs)

Name	End-group		*Boc*-Protected					Deprotected		
	α	ω	Hydrophobic group (mol %)	DP	M_n, ^{1}H NMR (g/mol)	Mn, GPC [1] (g/mol)	Đ, GPC [2]	Hydrophobic group (mol %)	DP	M_n, ^{1}H NMR (g/mol)
**HP1**	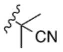	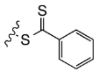	0	10.9	2,700	2,400	1.15	0	15.2	3,900
**HP2**	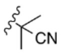	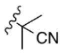	0	*n.d.*	*n.d.*	2,700	1.15	0	*n.d.*	*n.d.*
**HP3**	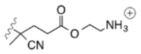	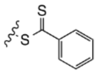	0	14.0	3,600	2,400	1.11	0	15.7	3,600
**CP1**	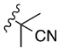	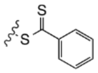	48.7	13.7	2,800	2,100	1.12	47.5	16.8	3,500
**CP2**	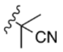	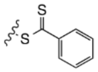	26.9	15.0	3,400	2,100	1.14	27.3	16.2	3,900
**CP3**	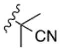	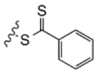	35.3	14.5	3,200	2,100	1.17	32.8	14.1	3,400
**CP4**	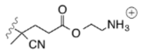	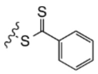	32.3	11.1	2,800	2,000	1.11	27.6	10.5	2,900
**CP5**	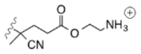	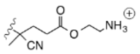	27.5	*n.d.*	*n.d.*	2,000	1.11	26.8	*n.d.*	*n.d.*

[1] The number average molecular weight (M_n) determined by GPC. The molecular weight calibration was based on poly(methyl methacrylate) standards.
[2] Dispersity (Đ) was calculated as M_w/M_n using M_w and M_n values determined by GPC.
n.d., not determined.

Supplementary Table S2: Growth inhibitory effect of synthetic biomimetic polymethacrylates (SBPs) on freshwater green algae *Chlamydomonas reinhardtii* (*CR*) and *Desmodesmus quadricauda* (*DQ*) and cyanobacteria *Synechococcus elongatus* (*SE*) and *Microcystis aeruginosa* (*MA*) based on optical density.

SBP		GREEN ALGAE						CYANOBACTERIA					
		Chlamydomonas reinhardtii (CR)			*Desmodesmus quadricauda (DQ)*			*Synechococcus elongatus (SE)*			*Microcystis aeruginosa (MA)*		
		**IC_{50}** [1] (μg mL^{-1})			**IC_{50}** (μg mL^{-1})			**IC_{50}** (μg mL^{-1})			**IC_{50}** (μg mL^{-1})		
		24 h [2]	48 h	72 h	48 h	72 h	96 h	24 h	48 h	72 h	48 h	72 h	96 h
		GEOMEAN (95% CI)	GEOMEAN (95% CI)	GEOMEAN (95% CI)	GEOMEAN 95% CI)	GEOMEAN (95% CI)	GEOMEAN (95% CI)	GEOMEAN (95% CI)	GEOMEAN (95% CI)	GEOMEAN (95% CI)	GEOMEAN (95% CI)	GEOMEAN (95% CI)	GEOMEAN (95% CI)
Homopolymer	**HP1**	**1.4** (0.8-2.4)	**1.0** (0.6-1.7)	**1.0** (06-1.7)	**9.7** (5.0-18.9)	**7.4** (4.1-13.2)	**5.1** (2.3-9.9)	**1.1** (0.7-1.6)	**0.9** (0.4-2.3)	**0.9** (0.5-1.6)	**1.1** (0.9-1.3)	**0.9** (0.6-1.5)	**1.0** (0.6-1.5)
	**HP2**	**0.6** (0.4-0.9)	**0.6** (0.2-1.5)	**0.6** (0.2-1.5)	**11.9** (6.0-23.5)	**6.4** (3.2-12.9)	**4.3** (2.8-6.7)	**0.9** (0.6-1.2)	**0. 9** (0.6-1.2)	**0.9** (0.6-1.2)	**1.3** (0.5-3.7)	**1.1** (0.5-2.5)	**1.0** (0.3-3.0)
	**HP3**	**0.9** (0.7-1.2)	**0.8** (0.7-0.8)	**0.7** (0.4-1.2)	**10.9** (4.8-24.7)	**8.9** (4.6-17.7)	**7.6** (5.6-10.1)	**0.9** (0.3-2.7)	**0.8** (0.4-1.4)	**0.9** (0.5-1.6)	**0.6** (0.3-1.5)	**0.9** (0.5-1.8)	**0.9** (0.4-2.1)
Copolymer	**CP1**	**1.0** (0.7-1.5)	**1.0** (0.8-1.2)	**1.1** (0.9-1.4)	**10.6** (6.1-17.4)	**9.1** (4.5-18.0)	**9.2** (5.1-16.4)	**1.1** (1.0-1.3)	**0.9** (0.6-1.8)	**1.1** (0.7-1.8)	**0.5** (0.1-2.5)	**0.5** (0.2-1.3)	**0.5** (0.2-1.1)
	**CP2**	**1.0** (0.4-2.1)	**1.0** (0.5-2.1)	**0.9** (0.7-1.0)	**4.6** (3.1-6.8)	**4.9** (4.2-7.7)	**5.2** (5.0-5.4)	**0.9** (0.7-1.2)	**0.7** (0.7-0.8)	**0.9** (0.7-1.3)	**0.2** (0.1-0.4)	**0.2** (0.1-0.6)	**0.3** (0.1-1.2)
	**CP3**	**1.4** (0.7-2.9)	**1.1** (0.6-1.8)	**1.1** (0.8-1.5)	**6.2** (3.5-10.8)	**5.9** (3.4-10.5)	**6.3** (2.5-15.9)	**1.2** (0.4-3.5)	**0.9** (0.5-1.8)	**0.8** (0.3-2.3)	**0.3** (0.2-0.5)	**0.3** (0.1-0.7)	**0.3** (0.1-1.2)
	**CP4**	**0.9** (0.5-1.6)	**0.9** (0.5-1.6)	**1.0** (0.4-2.5)	**12.8** (6.7-24.6)	**8.8** (5.6-13.7)	**7.6** (6.4-9.0)	**0.9** (0.7-1.2)	**1.2** (0.9-1.7)	**1.2** (0.9-1.5)	**0.3** (0.2-0.5)	**0.3** (0.1-0.8)	**0.3** (0.1-0.6)
	**CP5**	**1.0** (0.5-2.0)	**1.0** (0.5-2.0)	**1.0** (0.5-2.1)	**11.1** (4.3-28.6)	**8.1** (4.1-15.7)	**7.6** (34.5-13.0)	**1.0** (0.8-1.3)	**1.0** (0.8-1.3)	**1.1** (1.0-1.4)	**0.3** (0.1-0.6)	**0.3** (0.2-0.5)	**0.3** (0.1-0.6)

[1] The concentration of polymer causing a 50% inhibition of the algal or cyanobacterial growth. For each bioassay (independently repeated at least 3-times; $n \geq 3$), the IC_{50} value was determined, and the geometric mean with 95% confidence limits (CI; in parentheses) was calculated.

[b] Exposure time.

SUPPLEMENTARY FIGURES

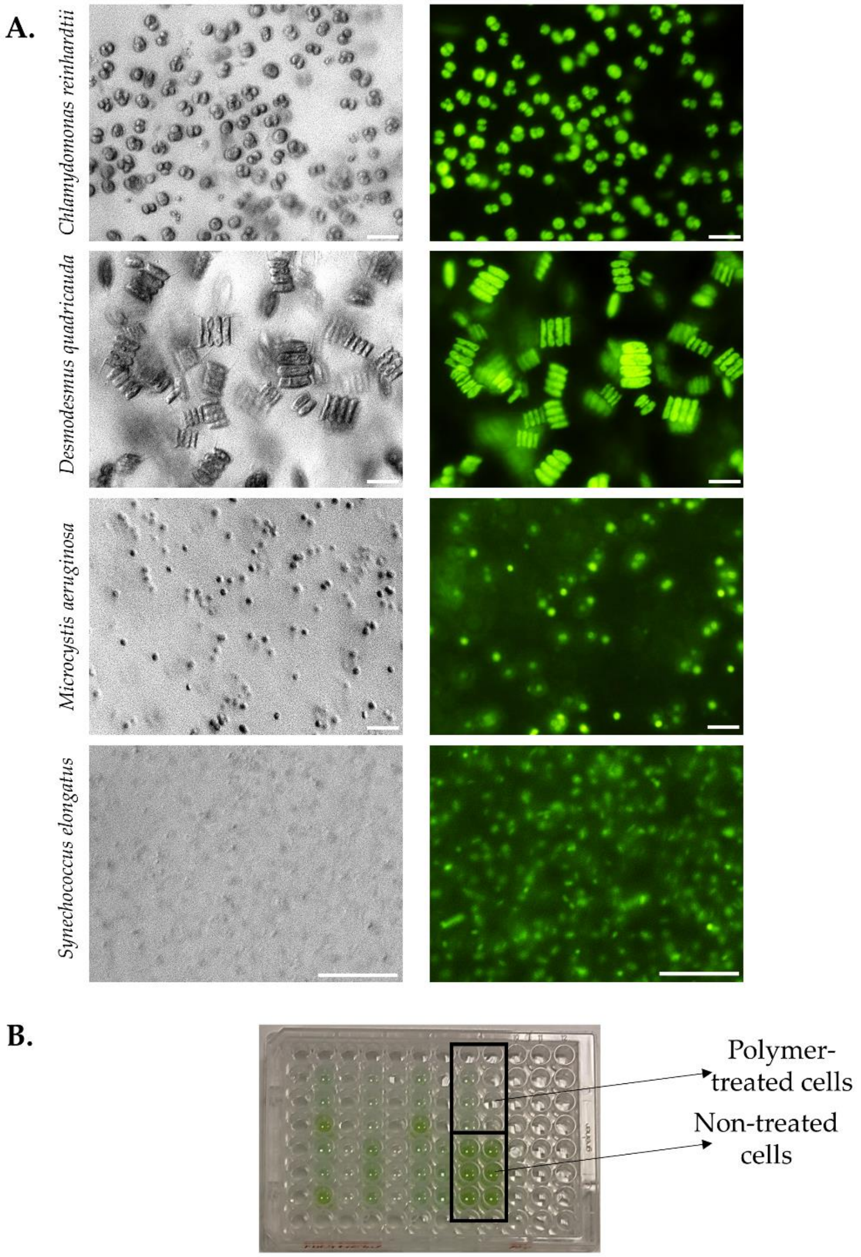

Supplementary Figure S1. Illustrative images of studied algal and cyanobacterial species (A.) and a 96-well plate at the end of growth inhibition and algicidal/ cyanocidal activity assays (B). A. Representative bright-field and fluorescence images of freshwater green algae *Chlamydomonas reinhardtii* (*CR*) and *Desmodesmus quadricauda* (*DQ*) and cyanobacteria *Microcystis aeruginosa* (*MA*) and *Synechococcus elongatus* (*SE*) taken by microscopy using upright fluorescence microscope Zeiss Axio Observer Z1 coupled to digital camera AxioCam 503 Mono (Zeiss, Jena, Germany). Non-stained samples were observed in the bright field or fluorescence regime using Zeiss filter set No. 05 (ex. 395-440 BP/em. 470 LP). Scale bar = 20 µm. **B.** A typical image of 96-well plate at the end of growth inhibition and algicidal/cyanocidal assays.

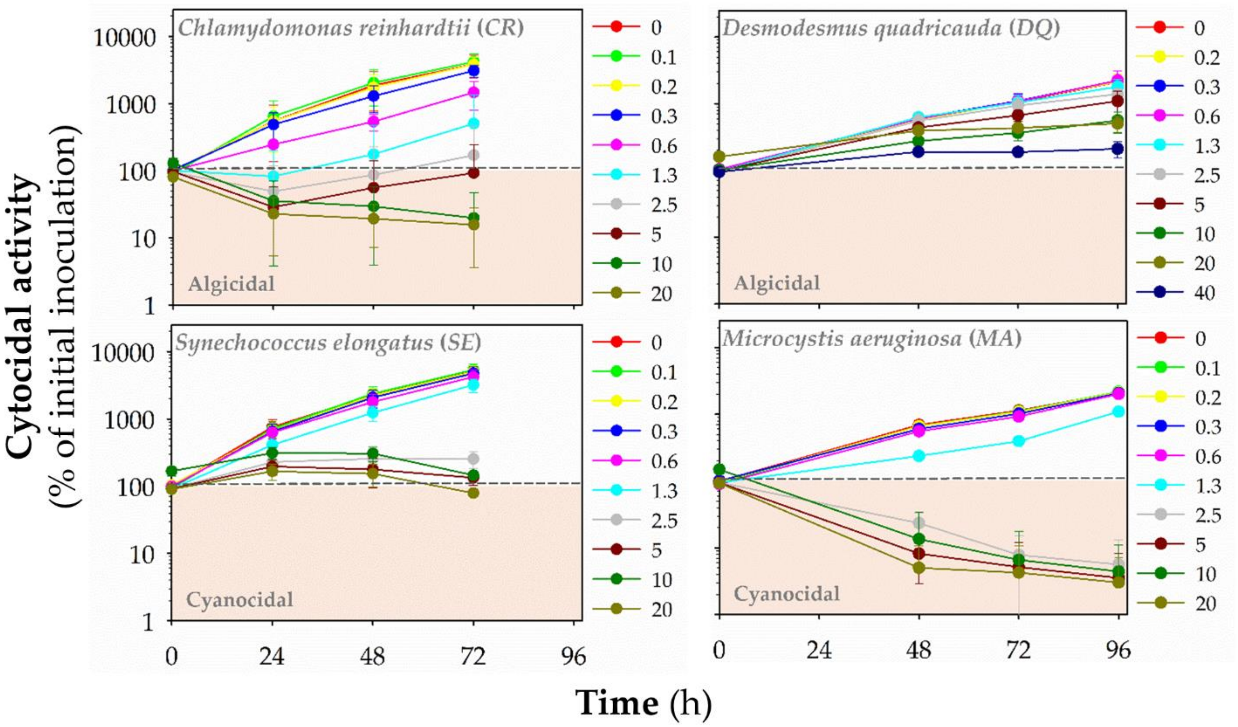

Supplementary Figure S2. The cytocidal activity of homopolymer HP2 against freshwater green algae *Chlamydomonas reinhardtii* (*CR*) and *Desmodesmus quadricauda* (*DQ*) and cyanobacteria *Synechococcus elongatus* (*SE*) and *Microcystis aeruginosa* (*MA*). The time- and concentration-dependent effects of the polymers in the range of concentrations of 0-40 µg mL^{-1} on the initial cell number (inoculum) was assessed by red fluorescence (λex = 485 nm/λex = 675 nm for algae, λex = 590 nm/λex = 675 nm for cyanobacteria) at different time points: 24-72-h (*CR*, *SE*) or 48-96h (*DQ*, *MA*). Data are expressed as a percentage of fluorescence units (RFUs) for initial inoculum and presented as means (±SD) of 3-5 independent experiments.

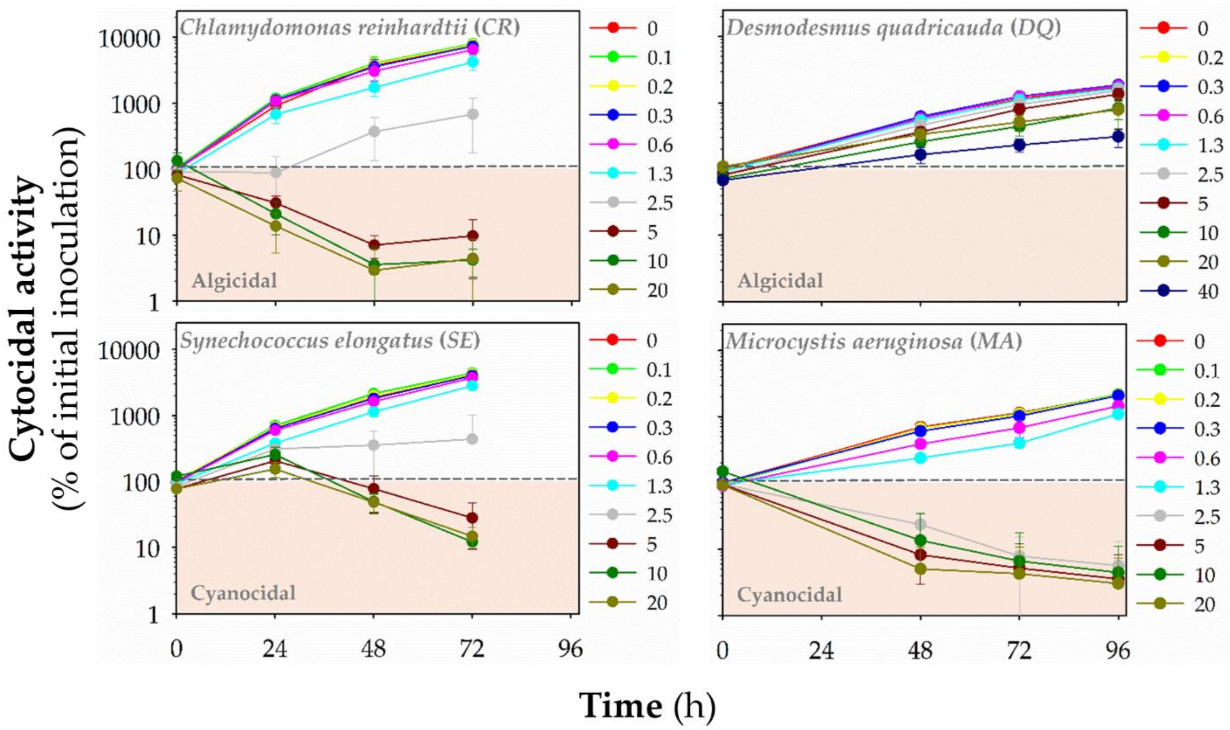

Supplementary Figure S3. The cytocidal activity of copolymer CP1 against freshwater green algae *Chlamydomonas reinhardtii* (*CR*) and *Desmodesmus quadricauda* (*DQ*) and cyanobacteria *Synechococcus elongatus* (*SE*) and *Microcystis aeruginosa* (*MA*). The time- and concentration-dependent effects of the polymers in the range of concentrations of 0-40 μg mL^{-1} on the initial cell number (inoculum) was assessed by red fluorescence (λex = 485 nm/λex = 675 nm for algae, λex = 590 nm/λex = 675 nm for cyanobacteria) at different time points: 24-72-h (*CR*, *SE*) or 48-96h (*DQ*, *MA*). Data are expressed as a percentage of fluorescence units (RFUs) for initial inoculum and presented as means (±SD) of 3-5 independent experiments.

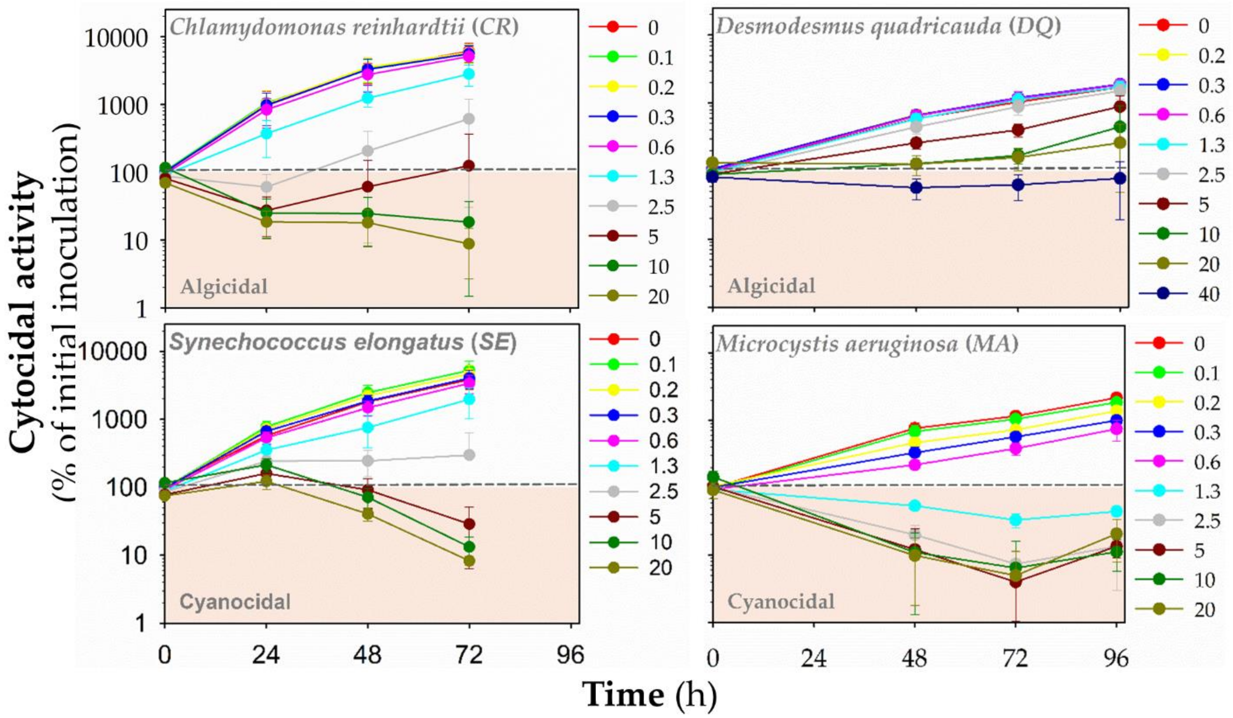

Supplementary Figure S4. The cytocidal activity of copolymer CP3 against freshwater green algae *Chlamydomonas reinhardtii* (*CR*) and *Desmodesmus quadricauda* (*DQ*) and cyanobacteria *Synechococcus elongatus* (*SE*) and *Microcystis aeruginosa* (*MA*). The time- and concentration-dependent effects of the polymers in the range of concentrations of 0-40 µg mL^{-1} on the initial cell number (inoculum) was assessed by red fluorescence (λex = 485 nm/λex = 675 nm for algae, λex = 590 nm/λex = 675 nm for cyanobacteria) at different time points: 24-72-h (*CR, SE*) or 48-96h (*DQ, MA*). Data are expressed as a percentage of fluorescence units (RFUs) for initial inoculum and presented as means (±SD) of 3-5 independent experiments.

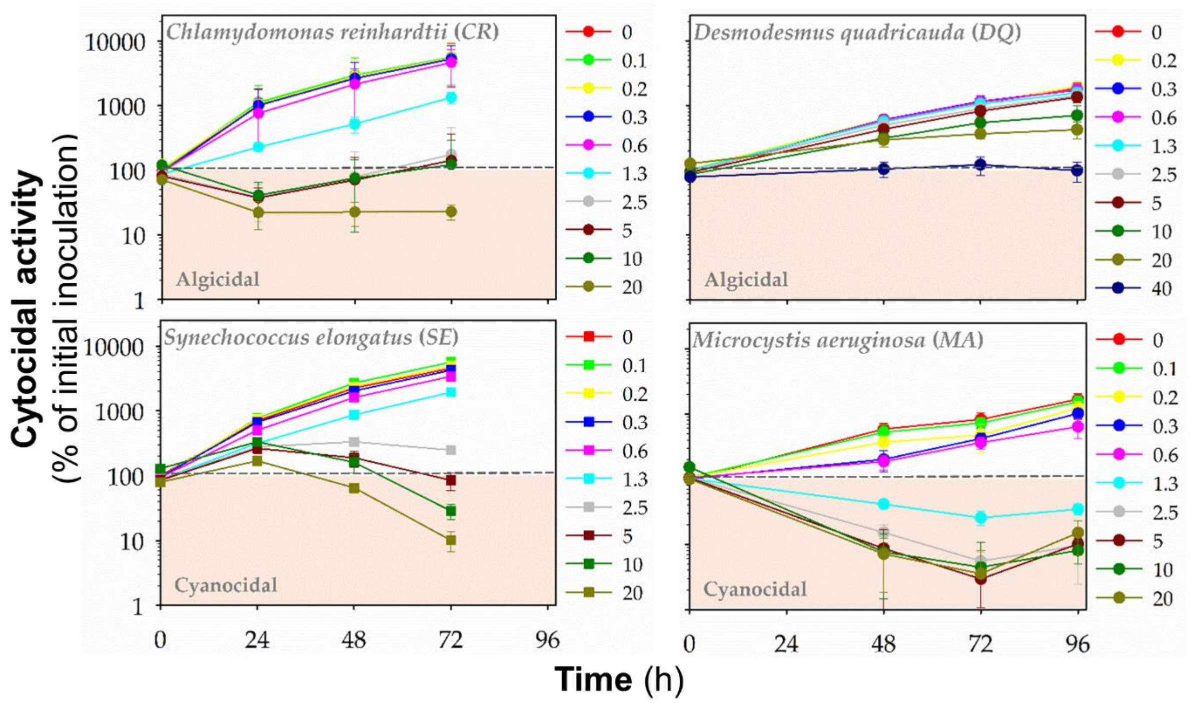

Supplementary Figure S5. The cytocidal activity of copolymer CP5 against freshwater green algae *Chlamydomonas reinhardtii* (*CR*) and *Desmodesmus quadricauda* (*DQ*) and cyanobacteria *Synechococcus elongatus* (*SE*) and *Microcystis aeruginosa* (*MA*). The time- and concentration-dependent effects of the polymers in the range of concentrations of 0-40 µg mL^{-1} on the initial cell number (inoculum) was assessed by red fluorescence (λex = 485 nm/λex = 675 nm for algae, λex = 590 nm/λex = 675 nm for cyanobacteria) at different time points: 24-72-h (*CR, SE*) or 48-96h (*DQ, MA*). Data are expressed as a percentage of fluorescence units (RFUs) for initial inoculum and presented as means (±SD) of 3-5 independent experiments.
